# Multiomics analysis of neutrophils in SLE: insights from adult and paediatric disease

**DOI:** 10.1093/cei/uxaf077

**Published:** 2025-12-09

**Authors:** Grace Filbertine, Isobel Kynoch, Genna A Abdullah, Lucy Gill, Rudi Grosman, Marie M Phelan, Zoe McLaren, Tawatchai Deekajorndech, Direkrit Chiewchengchol, Nattiya Hirankarn, Helen L Wright

**Affiliations:** Institute of Life Course and Medical Sciences, University of Liverpool, Liverpool, UK; Center of Excellence in Immunology and Immune-mediated Diseases, Department of Microbiology, Faculty of Medicine, Chulalongkorn University, Bangkok, Thailand; Institute of Life Course and Medical Sciences, University of Liverpool, Liverpool, UK; Institute of Life Course and Medical Sciences, University of Liverpool, Liverpool, UK; Institute of Life Course and Medical Sciences, University of Liverpool, Liverpool, UK; High-Field NMR Facility, Liverpool Shared Research Facilities (LivSRF), University of Liverpool, Liverpool, UK; Institute of Systems, Molecular and Integrative Biology, University of Liverpool, Liverpool, UK; High-Field NMR Facility, Liverpool Shared Research Facilities (LivSRF), University of Liverpool, Liverpool, UK; Institute of Systems, Molecular and Integrative Biology, University of Liverpool, Liverpool, UK; Department of Rheumatology, Liverpool University Hospitals NHS Foundation Trust, Liverpool, UK; Division of Nephrology, Department of Pediatrics, Faculty of Medicine, Chulalongkorn University, Bangkok, Thailand; Center of Excellence in Immunology and Immune-mediated Diseases, Department of Microbiology, Faculty of Medicine, Chulalongkorn University, Bangkok, Thailand; Center of Excellence in Immunology and Immune-mediated Diseases, Department of Microbiology, Faculty of Medicine, Chulalongkorn University, Bangkok, Thailand; Institute of Life Course and Medical Sciences, University of Liverpool, Liverpool, UK

**Keywords:** systemic lupus erythematosus, neutrophils, metabolomics, transcriptomics

## Abstract

Neutrophils contribute to systemic lupus erythematosus (SLE) pathogenesis through reactive oxygen species and neutrophil extracellular trap (NET) production, and increased apoptotic debris which causes autoantibody production and immune complex formation. These processes drive inflammation and tissue damage. The aim of this study was to perform integrated transcriptomic and metabolomic analyses comparing paediatric and adult SLE neutrophils. Adult (aSLE) and paediatric (jSLE) patient and healthy adult (HA) and juvenile (HJ) control neutrophils were subjected to RNAseq and ^1^H-NMR metabolomics. Univariate, multivariate and multiomics enrichment analyses were conducted in R and with ingenuity pathway analysis (IPA). Transcriptomic analysis revealed distinct gene expression profiles. Adult and juvenile SLE neutrophils were enriched for genes regulating interferon (IFN)-α/β signalling, neutrophil degranulation and NET signalling pathways (IPA, adj.*P*-value <0.01). Gene Ontology analysis revealed enrichment in cell cycle and interferon signalling in aSLE and angiogenesis and tissue-specific development in jSLE. Metabolomic profiling identified distinct metabolic alterations in aSLE, with a greater complexity of metabolic changes in jSLE. Multivariate PLS-DA demonstrated group discrimination, particularly in aSLE (balanced accuracy 80%, sensitivity 80%). Variable importance in the projection >1 metabolites were enriched in taurine/hypotaurine and amino acid metabolism in aSLE. Integrating transcriptomic and metabolomic data strengthened IFN-α/β signalling, neutrophil degranulation and NET signalling (adj. *P* < 0.001). Additional metabolic pathways uniquely down-regulated in aSLE included glutamate and glutamine metabolism, nucleotide biosynthesis and tryptophan catabolism (adj.*P*< 0.01). In summary, neutrophils from SLE patients, especially in jSLE, displayed complex transcriptomic and metabolic profiles, with aberrant IFN responses and neutrophil activation.

## Teaser

In this study we have taken a multiomics approach to study metabolism (NMR Metabolomics) and gene expression (RNA sequencing) in blood neutrophils from adults and children with systemic lupus erythematosus (SLE). We have compared the patient neutrophils with healthy control adult and child neutrophils using multiomics and bioinformatics. We have discovered gene and metabolite signatures that are unique to adult and pediatric SLE, as well as shared metabolites, genes and signalling pathway activation signatures such as interferon(IFN)-induced signalling and neutrophil degranulation.

## Graphical Abstract

**Graphical Abstract:**
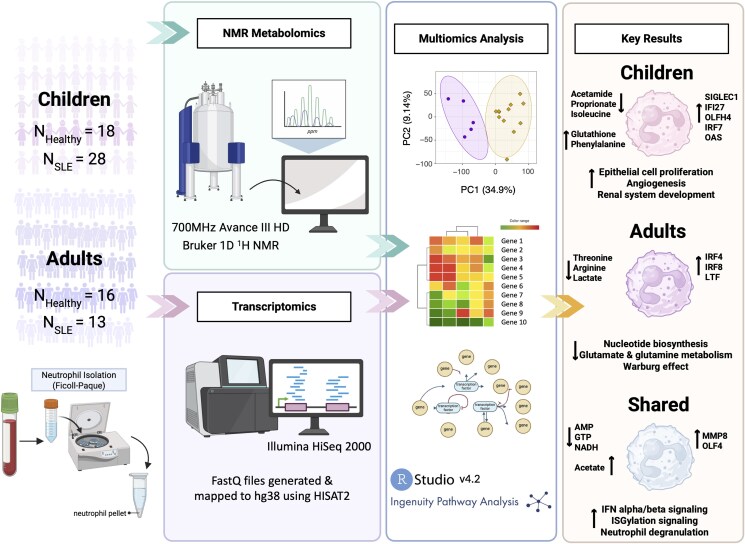
In this study, we have taken a multiomics approach to study metabolism (NMR Metabolomics) and gene expression (RNA sequencing) in blood neutrophils from adults and children with systemic lupus erythematosus (SLE). We have compared the patient neutrophils with healthy control adult and child neutrophils using multiomics and bioinformatics. We have discovered gene and metabolite signatures that are unique to adult and paediatric SLE, as well as shared metabolites, genes, and signalling pathway activation signatures such as interferon (IFN)-induced signalling and neutrophil degranulation.

## Introduction

Systemic lupus erythematosus (SLE) is a rare autoimmune disease characterized by multisystem involvement and a broad spectrum of clinical manifestations. Neutrophils contribute to systemic inflammation in SLE by the release of reactive oxygen species (ROS) and proteases, which damage host tissues. In addition, accumulation of apoptotic neutrophil debris and neutrophil extracellular trap (NET) fragments act as a substantial source of autoantigens leading to production of interferon alpha (IFNα) and the generation of autoantibodies. In lupus nephritis (LN), these mechanisms are particularly pronounced with deposition of immune complexes (IC) in the glomeruli causing a robust inflammatory response and ROS generation, resulting in significant renal damage and contributing to the observed pathogenesis [1].

In SLE, neutrophils display marked dysregulation, including increased rates of apoptosis and heightened inflammatory responses including cytokine production and mitochondrial dysfunction, all of which suggest a significant shift in cellular energy processing. The metabolic dysfunction observed in SLE neutrophils has profound implications for disease pathogenesis, particularly through the interconnected effects of impaired glucose uptake, heightened oxidative stress, and dysregulated formation of NETs. Studies on neutrophil transcriptomics in SLE have highlighted increased expression of interferon-stimulated genes (ISGs). However, the IFN signature does not manifest consistently across all SLE patients where a proportion are classified as IFN ‘low’, with significant implications for therapeutic targeting [2–4]. This intricate interplay between aberrant SLE neutrophils with immune complexes and type-I IFN underscores the importance of understanding the underlying dysfunction within neutrophils in individuals with SLE.

Recent advances in metabolomics have provided deeper insights into the metabolic alterations associated with human inflammatory diseases [5]. Among these, NMR and mass-spectroscopy have emerged as powerful tools for profiling metabolic changes, enabling the identification of potential disease biomarkers and novel therapeutic targets. While discriminating SLE and LN has been a major focus, a critical gap remains as none of these studies have investigated neutrophils. Given that SLE is an autoimmune disease driven by immune dysfunction, and neutrophils play a key role in its pathogenesis, the absence of neutrophil-focused metabolomics research represents a significant oversight. Exploring neutrophil metabolism could provide novel insights into their contribution to immune dysregulation in SLE, potentially revealing new biomarkers and therapeutic targets. By combining metabolic profiling with transcriptomics a multiomics approach was employed to enhance understanding of altered neutrophil activation in SLE. The aim of this study was to address this fundamental knowledge gap and provide critical insights into the unique phenotypic alterations in neutrophils associated with immune dysregulation in SLE, potentially uncovering new pathways for therapeutic intervention.

## Methods

### Ethical approval

This study was subject to ethical approval in Thailand and the UK. Recruitment of people with SLE and healthy controls in Thailand was approved by the Ethics Committee Institutional Review Board in the Faculty of Medicine, Chulalongkorn University, Thailand (IRB No.0551/65). Transfer of human tissue samples to from the UK to Thailand was approved by the University of Liverpool Central University Research Ethics Committee (No. 11635). Recruitment of people with SLE in the UK was approved by the NRES Committee North West (Greater Manchester West, UK, Ref: 11/NW/0206) under the Inflammatory Signalling Pathways study and by NRES West Midlands Solihull Research Ethics Committee (Ref 23/WM/0170) Pathways in Inflammation study. Recruitment of healthy controls in the UK was approved by the University of Liverpool Central University Research Ethics Committee (No. 10956). All study participants gave written informed consent in accordance with the Declaration of Helsinki. Clinical demographics of adult (aSLE) and juvenile SLE (jSLE) patients are shown in Supplementary Table S1 and details of prescription medications for each group of participants are shown in Supplementary Table S2. All paediatric samples (jSLE and healthy juvenile controls [HJ]) were recruited in Thailand. aSLE patients were recruited in the UK (*n* = 5) and Thailand (*n* = 13). Healthy adult (HA) controls were recruited in Thailand (*n* = 13) and the UK (*n* = 3). All samples were processed using the same protocol. HA and HJ participant demographics are shown in Supplementary Table S3.

### Neutrophil isolation

Human peripheral blood samples were obtained by venipuncture into lithium-heparin vacutainers. Neutrophils were isolated using Ficoll-Paque as previously described [6], a method that has been optimized and validated for NMR metabolomics analysis of neutrophils from small amounts of whole blood [7]. Contaminating erythrocytes were removed with hypotonic ammonium chloride lysis buffer and cells resuspended in RPMI-1640 media. Neutrophil purity was routinely >97% as confirmed with Wright Giemsa staining.

### Preparation of samples for NMR metabolomics

Neutrophils were pelleted by centrifugation at 1000 g at 25°C for 2 min and supernatant was aspirated. Cell pellets were heated at 100°C for 1 min prior to being snap-frozen using liquid nitrogen and stored at −80°C for further analysis.

### Metabolite extraction

Neutrophil metabolites were extracted using 50:50 v/v ice-cold HPLC-grade acetonitrile:water (ddH_2_O) as previously described [6, 8, 9], flash frozen in liquid nitrogen, lyophilized and stored at −80°C prior to analysis.

### NMR spectral acquisition and quality assurance of acquired spectra

Lyophilized metabolite pellets were resuspended in 200 μl of 100 mM deuterated sodium phosphate buffer pH 7.4 with 100 μM of TSP-d4 and 0.05% NaN_3_. Samples were vortexed for 20 s and centrifuged at 12 000 g for 1 min at 20°C, prior transferring into 3 mm NMR tubes for acquisition using 700 MHz Avance IIIHD Bruker NMR spectrometer equipped with TCI cryoprobe and chilled SampleJet^TM^ autosampler. Spectra were acquired using standard 1D ^1^H Carr-Purcell-Meiboom-Gill (CPMG; cpmgpr1d) spectra with 256 transients. All spectra quality were thoroughly evaluated to ensure they met the current best practice of Metabolomics Society [8, 10, 11] ensuring a robust consistency in phasing, baseline correction, peak alignment, chemical shift reference to TSP-d4 at 0 ppm, line width, and water suppression conditions for neutrophil intracellular metabolomic reporting. Following spectral acquisition and preliminary quality control in TopSpin v4.4.1, each sample underwent baseline correction using MestreNova (MNova) v15.1 (Mestrelab Research S.L., Santiago de Compostela, Spain) [12] accessed through nmrbox (nmrbox.nmrhub.org) [13] to address baseline distortions around residual water signal region (4.40–5.00 δ ppm) as observed in spectrum with weak signal due to smaller sample quantity [11]. Once quality-checked, all spectra passing QC in TopSpin v4.4.1, were uploaded into NMR Procflow v1.4 [14] for precise bin adjustment (ppm threshold) and further metabolite annotation.

### Annotation for neutrophil intracellular metabolomic profiling

Manual annotation of neutrophil metabolite peaks was performed as previously described [9]. A representative peak was selected for each metabolite annotated via correlation-based scoring (CRS) to remove redundancy in the dataset and reduce the number of variables for each NMR sample [15].

### Metabolomics statistical analysis

All data pre-processing and statistical analyses were performed in R (v4.2.0), including supervised and unsupervised multivariate analysis using the mixOmics package (v6.32.0) [16]. Experimental data were pre-processed using total area normalization and tested for normality with the Shapiro–Wilk test prior to statistical analyses. Univariate analysis was carried out by ANOVA with Tukey’s *post-hoc* analysis with application of an adjusted *P*-value of <0.05 using Benjamini–Hochberg (BH) false discovery rate (FDR) method. For multivariate analysis, the normalized data were Pareto scaled before applying principal component analysis (PCA) and partial least squares discriminant analysis (PLS-DA). The PLS-DA model was built upon a random selection of 70:30 (training:test) ratio with 10 randomly sampled model replicates each with a leave-one-out cross validation [17, 18]. As PLS-DA are supervised discriminatory models, performance metrics were calculated to determine model robustness in accordance with best practice [19]. Metrics of model performance (ability to predict SLE from healthy control) are measured in terms of specificity, sensitivity, and balanced accuracy whereby each model built was appraised for number of true positives (correct prediction or classification) and false positives (incorrect prediction or classification. It is noted that multiple methods exist for establishing performance of a PLS-DA model [20], for this study, sensitivity and specificity were selected as long established methods that are widely used and understood (see for further explanation, see [21]). Variable importance in the projection (VIP) scores were derived for each PLS-DA model to establish which metabolites were most influential within the model to discriminate between SLE and healthy control. As metabolites with a VIP score greater than 1 exert an above average influence, these metabolites were considered important in distinguishing between groups.

### RNA extraction

Total RNA was extracted from isolated neutrophil pellets using Trizol as previously described [22]. Isolated RNA was then further purified using Qiagen RNeasy kit (mRNA) or Qiagen miRNeasy kit (miRNA) including an on-column DNase digestion step. Total RNA was snap frozen in liquid nitrogen prior storing at −80°C. RNA integrity was measured using the Agilent 2100 fragment analyser. Samples with RNA integrity (RIN) > 6.8 passed QC and processed for sequencing.

### RNA sequencing and statistical analysis

Total RNA was enriched with poly-A selection for mRNA. cDNA libraries were generated using Illumina TruSeq RNA Sample Preparation Kit. Libraries sequenced on the DNBseq platform by BGI, Hong Kong (https://www.bgi.com). Sequencing generated 100 base pair (bp) paired end reads for each sample. Raw reads were mapped to the current version of the human genome (hg38) using HISAT2 [23]. Read counts were generated using featureCounts and statistical analysis was carried out using edgeR package v3.40.2 [24] applying the weighted trimmed mean of M-values (TMM) normalization and an FDR-adjusted *P*-value correction. Differentially expressed genes (DEGs) were identified by applying an FDR-adjusted *P*-value threshold of <0.05. DEGs with fold change (FC) thresholds of ≥1.5 (upregulated) and ≤−1.5 (downregulated) between sample groups and/or genes with an FDR <0.05 between sample groups were selected for further analysis.

### qPCR analysis

cDNA was synthesized using the Superscript III assay kit (Thermo Fisher) for mRNA and the miRCury LNA assay kit (Qiagen) for miRNAs. Quantitative PCR analysis of mRNA was performed using QuantiNova SYBR Green PCR kit (Qiagen) on a Roche 480 Lightcycler. Target gene expression (IFI44L, IFIT1B, OAS1, LCN2, LTF, MMP8, MPO) was quantified against B2M housekeeping gene using validated primers. Quantitative PCR analysis of miRNA was performed using miRCURY LNA SYBR Green PCR kit (Qiagen) on a Roche 480 Lightcycler. Target gene expression (miR-182-5p, miR-199a-5p, miR-96a-5p, and miR-183a-5p) was measured using validated miRCURY LNA primers. The reference gene for miRNA was computed by RefFinder, which determined miR-16-5p as the most stable. Target gene expression was calculated using the Pfaffl method [25].

### Bioinformatics analysis

Gene ontology biological process enrichment analysis of RNAseq up-regulated DEGs was performed using the R package clusterProfiler (v4.6.2) [26] applying a *P*-value cutoff of 0.05 with Benjamini–Hochberg *P*-value adjustment. Canonical pathway enrichment analysis and upstream regulator analysis of RNAseq DEGs was performed using IPA (QIAGEN Inc., https://digitalinsights.qiagen.com/IPA) [27]. Functional enrichment analysis of metabolites was performed using MetaboAnalyst (v6.0, https://www.metaboanalyst.ca) [28] using the enrichment analysis module incorporating key metabolites identified through PLS-DA with a VIP score >1. Enrichment analysis was run against the KEGG database containing 80 human metabolic pathways (December 2023) and the blood disease signatures metabolite database (480 metabolite sets derived from human blood samples). The analysis employed FDR correction to adjust for multiple comparisons and were ranked based on their statistical significance (−log(p) values). Multiomics analysis was performed in IPA using a combined list of RNAseq DEGs and metabolites with >1.5-fold increase/decrease between comparisons. Heatmaps were generated in R using normalized data to visualize metabolite and gene variations across different groups. Data were log2-transformed and hierarchical clustering was applied to both metabolites and samples using Ward’s method to group similar patterns based on Euclidean distance.

## Supplementary Material

uxaf077_Supplementary_Data

## Figures and Tables

**Figure 1:**
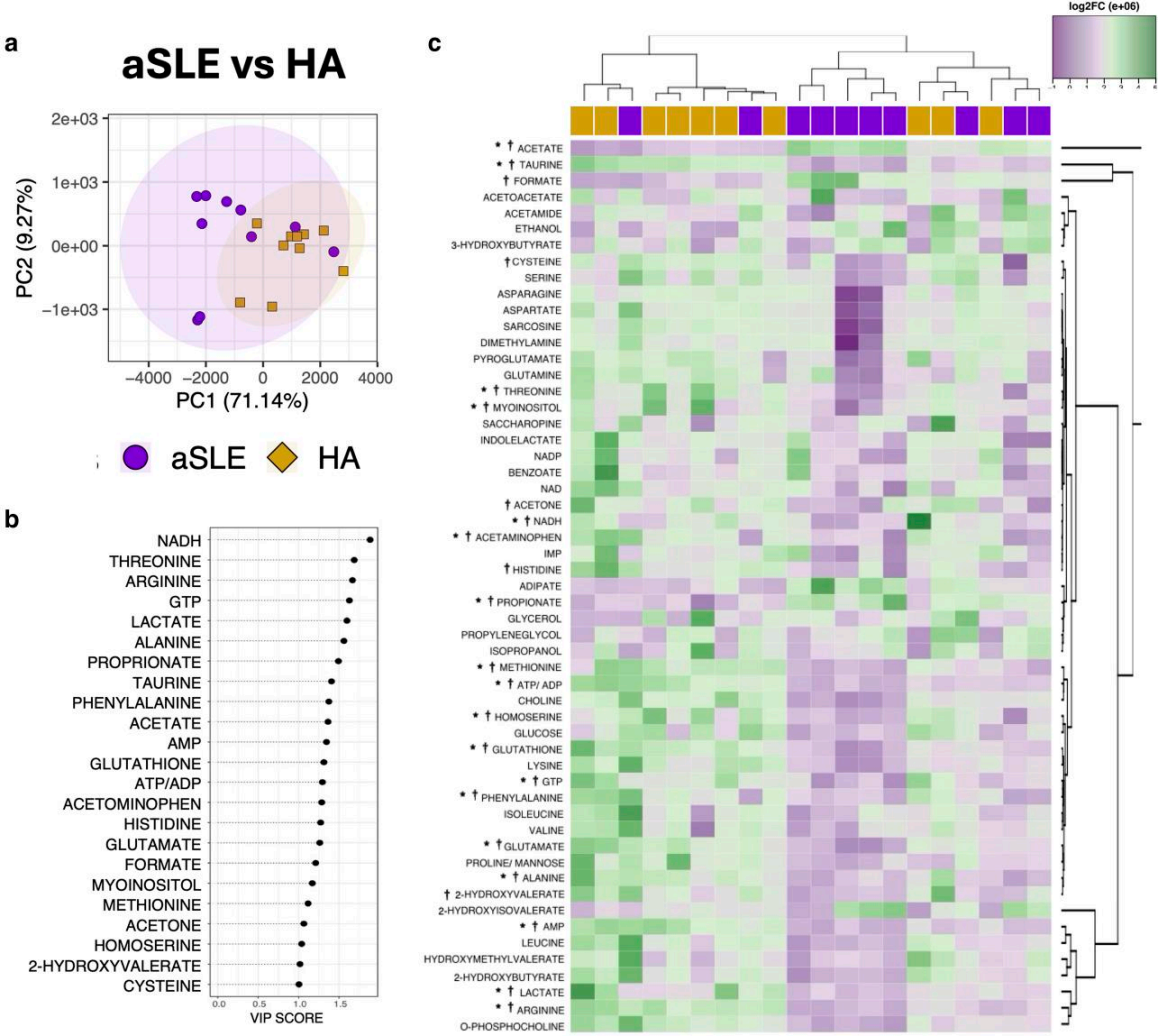
NMR metabolomics analysis of aSLE neutrophils. (a) PCA of aSLE (*n* = 10, purple) vs HA (*n* = 10, orange) neutrophil metabolomes. (b) VIP metabolites identified by multivariate PLS-DA of aSLE vs HA neutrophils. (c) Heatmap of all metabolites identified, with metabolites significant by univariate (*) and/or multivariate (†) analysis highlighted.

**Figure 2:**
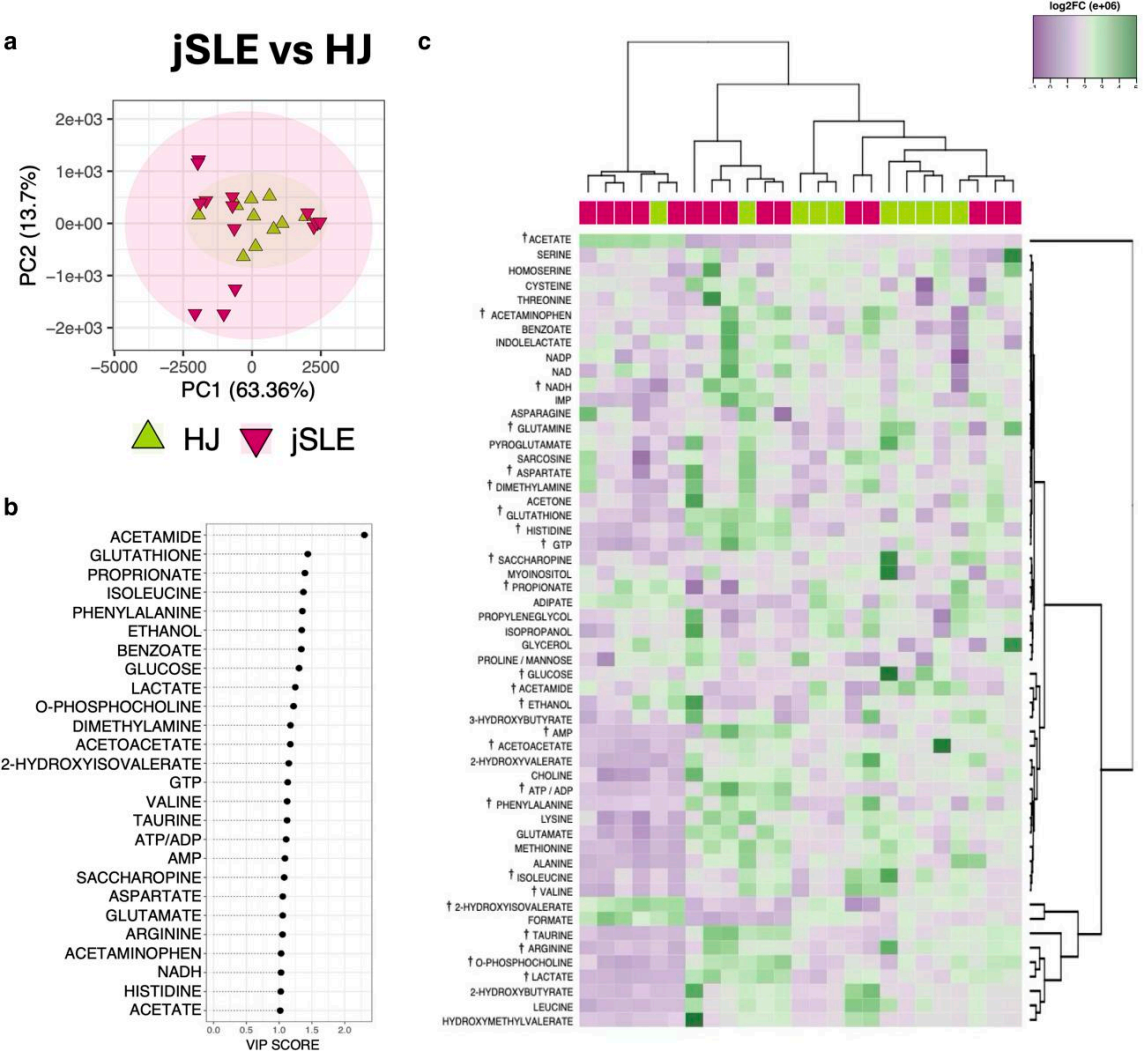
NMR metabolomics analysis of jSLE neutrophils. (a) PCA of jSLE (*n* = 15, pink) vs HJ (*n* = 10, green) neutrophil metabolomes. (b) VIP metabolites identified by multivariate PLS-DA of jSLE vs HJ neutrophils. (c) Heatmap of all metabolites identified, with metabolites significant by multivariate (†) analysis highlighted.

**Figure 3:**
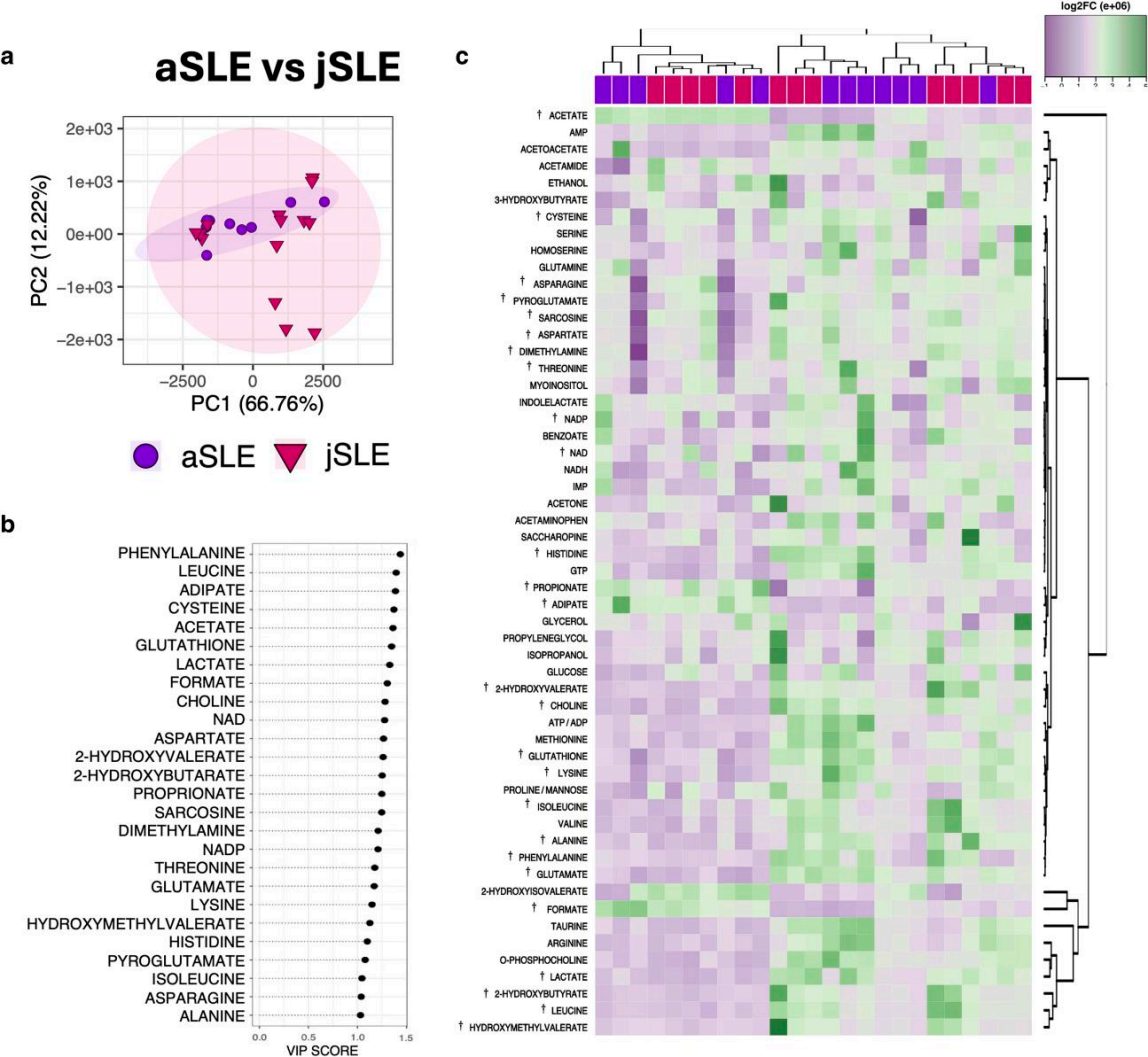
NMR metabolomics analysis of aSLE vs jSLE neutrophils. (a) PCA of aSLE (*n* = 10, purple) vs jSLE (*n* = 15, pink) neutrophil metabolomes. (b) VIP metabolites identified by multivariate PLS-DA of aSLE vs jSLE neutrophils. (c) Heatmap of all metabolites identified, with metabolites significant by univariate (*) and/or multivariate (†) analysis highlighted.

**Figure 4:**
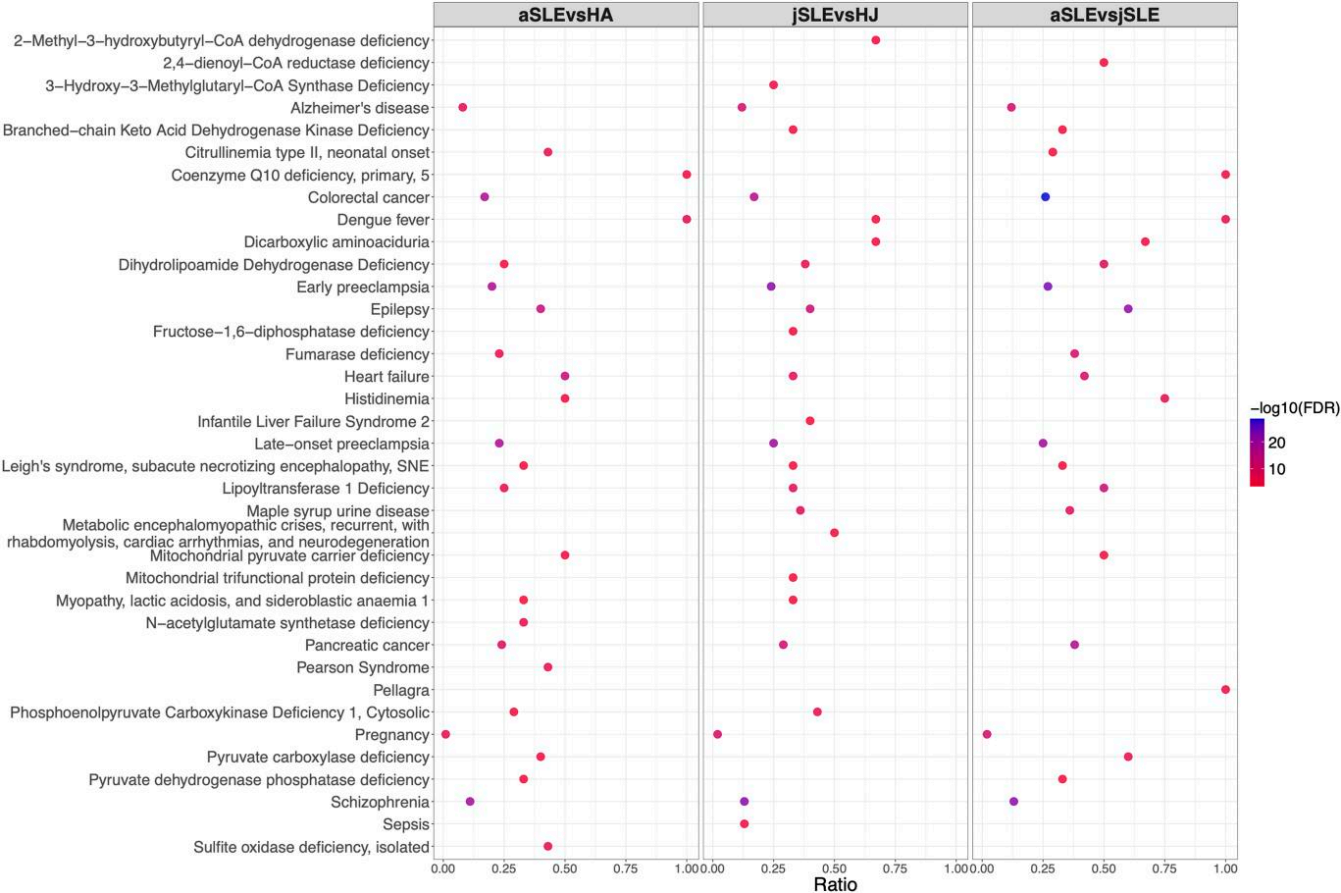
Metabolic pathway enrichment analysis of neutrophil metabolites. Pathway enrichment ratio and -log10(FDR) *P* value indicated for aSLE vs HA, jSLE vs HJ, and aSLE vs jSLE comparisons.

**Figure 5:**
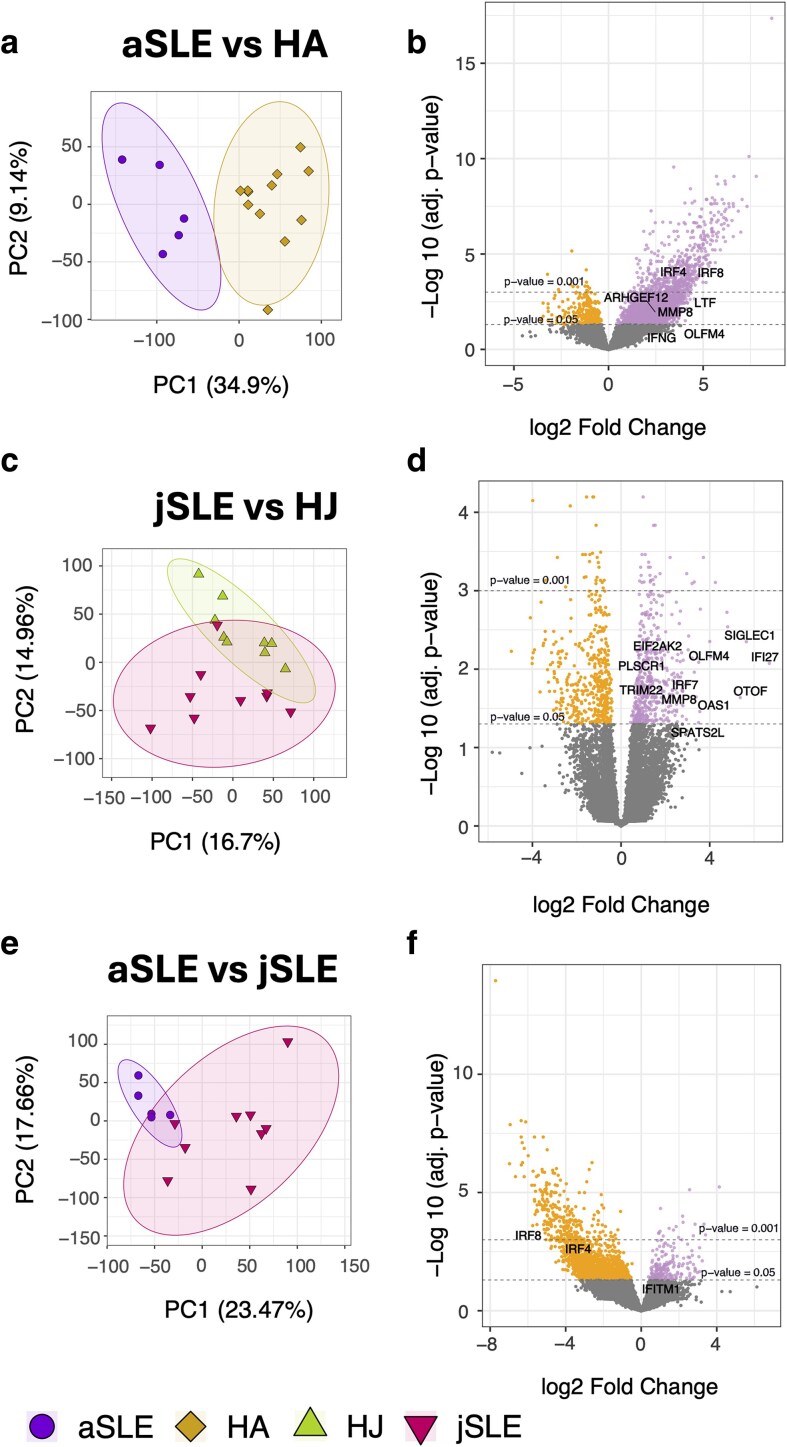
RNAseq analysis of SLE neutrophils. (a) PCA and (b) volcano plot of aSLE (*n* = 5) vs HA (*n* = 12) neutrophil transcriptomes. (c) PCA and (d) volcano plot of jSLE (*n* = 9) vs HJ (*n* = 8) neutrophil transcritpmes. (e) PCA and (f) volcano plot of aSLE (*n* = 5) vs jSLE (*n* = 9) neutrophil transcriptomes. Volcano plots showing genes significantly up-regulated (purple) and down-regulated (orange) (adj. *P* < 0.05). Neutrophil granule protein genes and interferon stimulated genes are highlighted by name.

**Figure 6:**
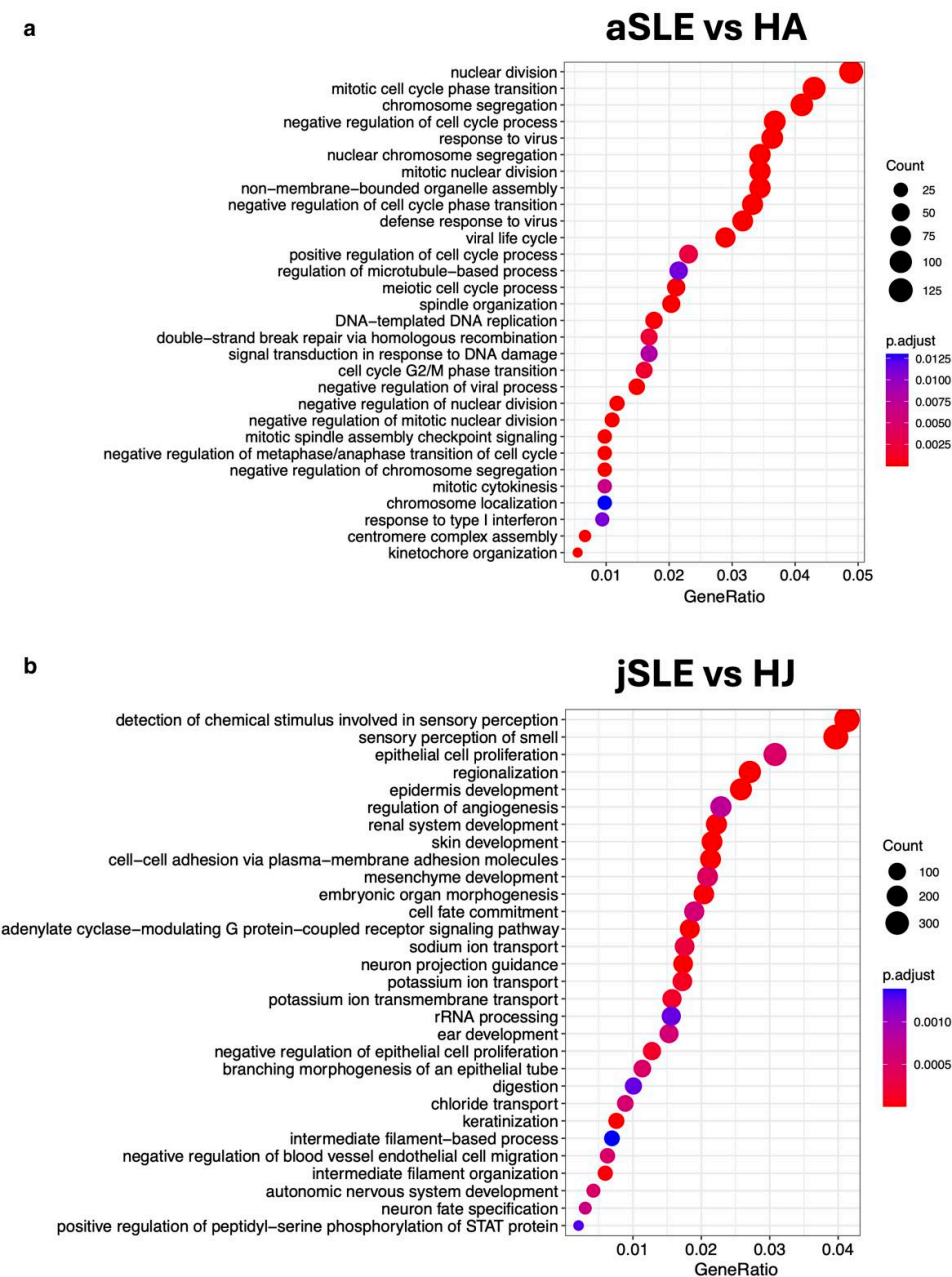
Gene ontology enrichment analysis of neutrophil genes expressed in SLE. KEGG analysis of significant gene ontology biological process enrichment in (a) aSLE vs HA and (b) jSLE vs HJ neutrophils.

**Figure 7:**
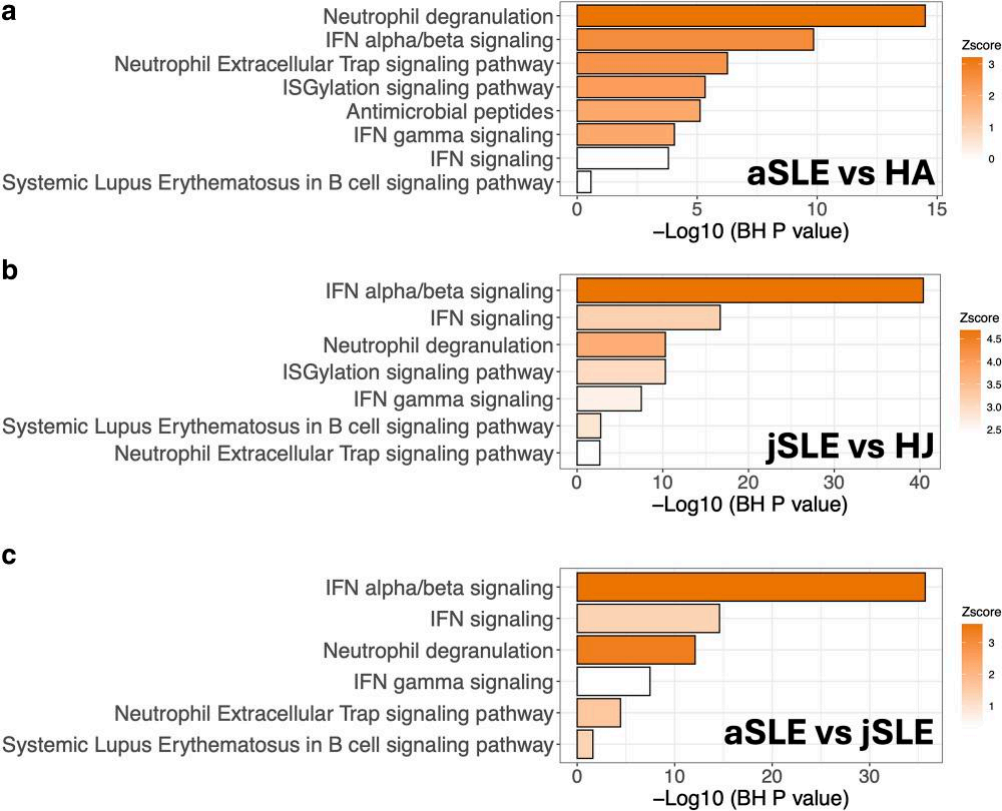
Pathway enrichment analysis of neutrophil genes expressed in SLE. IPA canonical pathway enrichment analysis of significant genes expression in (a) aSLE vs HA, (b) jSLE vs HJ and (c) aSLE vs jSLE neutrophil transcriptomes.

**Figure 8:**
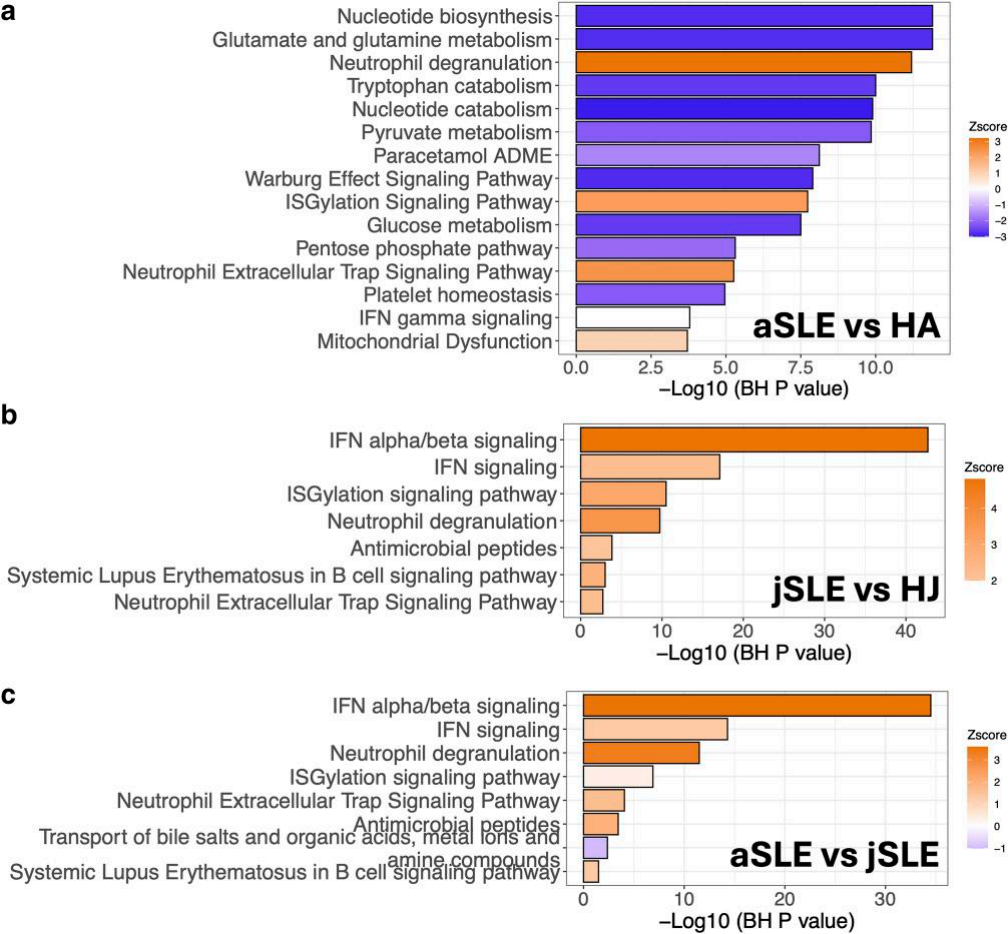
Multiomic pathway enrichment analysis using IPA. Multiomic analysis of significant neutrophil metabolites and genes using IPA for (a) aSLE vs HA, (b) jSLE vs HJ and (c) aSLE vs HJ. Z score indicates activation (orange) or inhibition (blue) of a signalling pathway.

**Figure 9:**
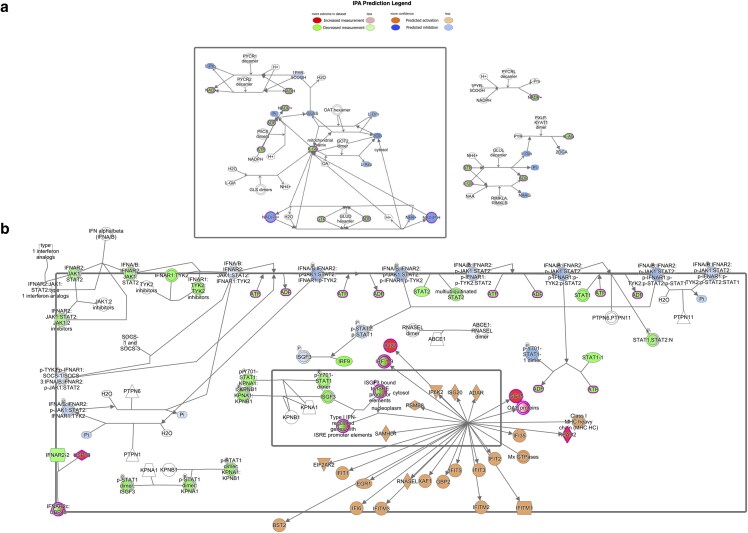
IPA signalling pathways enriched in SLE. (a) Glutamate and glutamine metabolism pathway. (b) Type I IFN (IFN alpha/beta) signalling pathway. Red, observed up-regulated gene/metabolite; green, observed down-regulated gene/metabolite; orange, predicted up-regulated gene/metabolite; blue, predicted down-regulated gene/metabolite; grey, observed gene/metabolite with no difference in abundance; white, not observed gene/metabolite.

## Data Availability

All metabolomics data including processing steps, parameters, and metabolite annotations and CRS representative peak selections are deposited in the European Bioinformatics Institute (EBI) open repository for metabolomics (www.ebi.ac.uk/metabolights) with the ID number MTBLS6259 [29]. The raw RNA sequencing data reported in this manuscript have been deposited in the NCBI Gene Expression Omnibus (GEO) [30] and are accessible through GEO Series accession number GSE293224.

## Results

### Neutrophil energy metabolism is dysregulated in SLE

In order to measure metabolite abundance in SLE and healthy neutrophils, we performed ^1^H 1D NMR metabolomics of neutrophil polar metabolite extracts using our previously optimized protocols [6, 7]. We identified 224 metabolite peaks across the whole spectrum, comprising 57 metabolites. Representative metabolite peaks were selected for analysis using CRS as previously described [9, 15] (Supplementary Table S4). Analysis of adult SLE (aSLE) and HA control neutrophil metabolites revealed distinct metabolite profiles. PCA scores results indicate that both PC1 and PC2 together explain 80.41% of the variance in aSLE vs HA (Fig. 1a). Univariate analysis of aSLE vs HA metabolites identified 18 significant (adj.*P*-value < 0.05) metabolites (Supplementary Table S5). Notably, propionate and acetate were elevated in aSLE vs HA. Supervised multivariate analysis (PLS-DA) demonstrated good performance. A 1-component model of aSLE vs HA had a balanced accuracy of 80% and exhibited high sensitivity (98%) and specificity (80%). Beyond model performance, the PLS-DA model highlighted influential metabolites (VIP) discriminating aSLE from healthy controls. In the aSLE vs HA comparison, the model detected 23 metabolites with a VIP score >1 (Fig. 1b). Interestingly, 19 of these metabolites were also identified as statistically significant (adj.*P*-value < 0.05) through univariate analysis, demonstrating strong agreement between the two approaches. A clear distinction between aSLE and HA metabolites was visible by heatmap (Fig. 1c). In aSLE, acetate, adipate, and propionate were notably elevated. Conversely, ATP, ADP, NADP+, AMP, taurine, glutamate, glutathione, glucose, lactate, choline, and several amino acids (leucine, isoleucine, valine, alanine, serine, cysteine, methionine, homoserine, phenylalanine, and arginine) were lower in sSLE compared to HA.

PCA scores results for juvenile SLE (jSLE) vs HJ controls indicated that PC1 and PC2 explained 77.06% of the variance (Fig. 2a). No significant metabolite differences were observed by univariate analysis of the paediatric cohort (jSLE vs HJ). Notably the jSLE samples separated into three distinct clusters. These did not correlate to any clinical parameters, such as disease activity, kidney involvement, medication, or to demographics such as age or sex. All paediatric samples were of South Asian ethnicity. A 2-component PLS-DA model demonstrated balanced accuracy of 59% with low sensitivity (48%) and moderate specificity (72%). The model identified 26 metabolites with VIP > 1 (Fig. 2b), although none of these metabolites were statistically significant by univariate analysis. Two component PLS-DA models were extremely varied in classification metrics for this cohort (ROCs between 0.5 and 0.85, Supplementary Figure S1) and as such were not deemed sufficiently robust. Taking into account the influential metabolites within PLS-DA models of moderate quality (ROC >0.85), a non-supervised PCA plot was built using only those selected variables and whilst some samples of jSLE clustered separate to the HJ controls there was still sample overlap between the two patient groups (Supplementary Figure S2). The heatmap of jSLE vs HJ metabolites revealed two distinct clustering patterns (Fig. 2c), further highlighting metabolic heterogeneity within the juvenile group. A cluster of 5 jSLE and 1 HJ displays an increase in acetate, adipate, 2-hydroxyisovalerate, and formate, alongside lower AMP, ATP, ADP, choline, and several amino acids (phenylalanine, lysine, glutamate, methionine, alanine, isoleucine, valine, leucine, arginine, taurine). A second cluster (jSLE = 5, HJ = 1) displayed the complete opposite metabolic profile compared to cluster 1.

PCA scores results for the comparison of adult and juvenile SLE groups indicated PC1 and PC2 explained 78.98% of the variance between aSLE and jSLE metabolomes (Fig. 3a). There were no significant metabolite differences between aSLE and jSLE by univariate *t*-test. For aSLE vs jSLE a 1-component PLS-DA model had a balanced accuracy of 71% with low sensitivity (55%) but high specificity (86%). The jSLE vs HJ model identified 26 metabolites with VIP > 1 (Fig. 3b), indicating their importance in differentiating aSLE and jSLE. Several metabolites were notably higher in jSLE, including leucine, choline, NAD, NADP+, 2-hydroxybutyrate, sarcosine, lysine, hydroxymethylvalerate, pyroglutamate, and asparagine, while adipate was the only metabolite elevated in aSLE. These differences can be observed in the heatmap (Fig. 3c).

Pathway Enrichment Analysis in MetaboAnalyst, based on key metabolites with a VIP score >1 from PLS-DA, ranked the top 25 metabolite pathways (Fig. 4) using the KEGG database (80 human metabolic pathways) and disease-specific datasets (480 metabolite sets from human blood samples) [31, 32]. Interestingly, SLE or localized lupus erythematosus (e.g. DLE) did not appear among the top 25 disease-associated pathways. Disease pathways associated with early and late pre-eclampsia were enriched in all SLE comparisons. Many of the dysregulated immune pathways in pre-eclampsia are similar to SLE, including activated neutrophils, oxidative stress, and endothelial dysfunction. In terms of metabolic pathways, aSLE was associated with taurine and hypotaurine metabolism, glutathione metabolism, and glyoxylate and dicarboxylate metabolism, while jSLE showed enrichment in arginine biosynthesis, histidine metabolism, and alanine, aspartate, and glutamate metabolism. The comparison between aSLE and jSLE highlighted potential changes in valine, leucine and isoleucine biosynthesis, alanine, aspartate and glutamate metabolism, and glutathione metabolism. Additional pathways included glycolysis/gluconeogenesis, purine metabolism (only in comparisons with healthy controls), lysine metabolism (absent in aSLE vs HA), and several other amino acid metabolism pathways among the top 25. Taken together, the metabolomics results indicate skewing of metabolism in aSLE, in particular with regards to a decrease in glutathione and amino acid metabolism as well as a down-regulation of energy metabolites. However, the metabolic profile of jSLE neutrophils is more heterogeneous and does not reflect the metabolic changes observed in adult disease in all patients.

### Gene expression in SLE neutrophils is characterized by an up-regulated interferon-induced signature and expression of genes for neutrophil granule proteins

We next used bulk RNAseq to analyse the transcriptomic differences between adult and paeditric SLE neutrophils compared to their respective healthy controls. PCA scores plot of aSLE vs HA neutrophil transcriptomes revealed complete separation of aSLE and HA samples along PC1 (Fig. 5a, PC1 34.9% of variance). We did not observe any sub-clustering of UK and Thai HA samples by PCA. Volcano plot (Fig. 5b) showed significant up-regulation of genes, in particular those associated with ISGs and genes encoding neutrophil granule proteins (NGPs). Notable genes such as MMP8, LTF, IRF4 and IRF8 are over expressed in aSLE neutrophils. PCA scores plot of jSLE vs HJ neutrophil transcriptomes showed some separation of samples along PC2 (Fig. 5c). We did not observe the sub-clusters that were observed in metabolomics analysis when comparing jSLE neutrophil transcriptomes. Volcano plot (Fig. 5d) revealed strong up-regulation of ISGs and NGPs in jSLE neutrophils. Key markers like SIGLEC1, OTOF, and IRF7 were significantly up-regulated in jSLE vs HJ transcriptomes. When comparing aSLE with jSLE, there was clear separation of populations by PCA scores plot (Fig. 5e), and a distinct gene expression pattern was notable by volcano plot (Fig. 5f). Notably, jSLE were characterized by the up-regulation of more ISGs compared to NGPs, and therefore a stronger interferon-induced signature in jSLE neutrophils compared to aSLE neutrophils. Although a broader upregulation of ISGs was evident in jSLE compared to aSLE, this was most pronounced within the IFIT, IFITM, and OAS families, with IFITM1 showing significantly higher expression in jSLE. We confirmed increased expression of ISGs (IFI44L, IFIT1B, and OAS1) in a validation cohort of aSLE patients recruited in the UK using qPCR but were unable to validate increased NGPs (MMP8, MPO, LCN2, and LTF) in these patients (Supplementary Figure S3a) likely due to low n-numbers (*n* = 5–6) which were insufficient to capture the heterogeneity in ISG and NGP expression between interferon ‘high’ and interferon ‘low’ SLE patients.

Heatmap analysis revealed distinct transcriptional profiles of ISGs (Supplementary Figure4a–c) and NGPs across the study groups (Supplementary Figure S4d–f). A total of 7 genes were significantly differentially expressed between aSLE and HA, while 12 genes were identified as significant between jSLE and HJ. Interestingly, consistent with observations from other studies, a subset of patients exhibited an IFN ‘low’ signature. Furthermore, in the HA, samples from Thailand we noted a subset of four individuals displaying an IFN ‘high’ signature, which was not found in HA samples collected in the UK, despite these participants meeting the enrollment criteria for healthy adult controls. In addition to ISGs, several neutrophil-specific genes were markedly upregulated in adult and juvenile SLE patients compared to their healthy counterparts. These included MMP8, CEACAM6, DEFA3, DEFA4, OLFM4, MPO, and LTF, all of which were elevated in both aSLE and jSLE groups.

Gene Ontology Biological Process (GOBP) enrichment analysis of DEGs using clusterProfiler revealed distinct patterns of functional enrichment in aSLE and jSLE neutrophils (Fig. 6). GOBP analysis in aSLE revealed significant enrichment of processes related to cell cycle dysregulation, including nuclear division, chromosome segregation, and mitotic cell cycle phase transition (Fig. 6a). Additional enrichment was observed in antiviral defence mechanisms and immune-related pathways, including defence response to virus, response to type I interferon, and negative regulation of cell cycle processes. For jSLE, the enrichment analysis showed a focus on developmental processes, such as epithelial cell proliferation, skin development, renal system development, and mesenchyme development, as well as processes related to sensory perception of smell and cell–cell adhesion via plasma membrane adhesion molecules (Fig. 6b). Several immune-related pathways, including regulation of angiogenesis and positive regulation of STAT phosphorylation, were also detected.

IPA was also applied to map these upregulated and downregulated genes to canonical pathways in the IPA knowledge base. The comparisons between aSLE and HA (Fig. 7a), and jSLE and HJ (Fig. 7b), produced very similar results which were enriched for canonical pathways relating to neutrophil degranulation, IFN-*α/β* Signalling, NET Signalling, ISGylation Signalling, and IFN Gamma Signalling (all adj. *P*-value < 0.05), underscoring the prominent role of innate immune responses, particularly neutrophil activity and IFN-related pathways in both aSLE and jSLE. Additional pathways of interest were identified but lacked sufficient activation z-scores to determine their functional direction in the analysis, including IFN signalling (aSLE vs HA), IFN*γ* signalling (aSLE vs jSLE, Fig. 7c), and SLE in B Cell Signalling Pathway (aSLE vs HA).

IPA upstream regulator analysis identified several key transcriptional regulators and microRNAs with significant roles in lupus pathogenesis, neutrophil function, and IFN signalling, aligned to previous analysis using DEGs. Among these, known transcription factors contributing to type I and type II IFN responses which emerged as highly activated regulators in all analysis among aSLE, jSLE, and their respective healthy controls as well as comparison among SLE across different age groups, such as STAT1, STAT2, IRF3, IRF7, and IRF1 (all adj. *P*-value < 0.05). Another notable set of activated upstream regulators identified includes CEBPE, which is specific to aSLE vs HA, CEBPA (specific to jSLE vs HJ), SPI1, and NONO (all adj. *P*-value < 0.05), all of which are critical to neutrophil biology and potentially contributed to the aberrant neutrophil responses observed in SLE. Furthermore, the upstream regulator analysis predicted gene regulation by microRNAs, particularly miR-199a-5p, miR-182-5p, as well as the small non-coding RNAs miR-183 and miR-96 (all adj. *P*-value < 0.05). We measured expression of these miRNAs in HA and aSLE neutrophils by qPCR, and whilst there was a trend towards elevation of these miRNAs in SLE, this did not reach statistical significance (Supplementary Figure S3b) likely for reasons stated above.

Taken together the results of the RNAseq analysis indicate a strong up-regulation of genes relating to neutrophil activation, DNA reorganization, and response to Type-1 and Type-II interferons in both adult and juvenile SLE. Similar activation pathways are observed in both paediatric and adult disease, with a stronger expression of ISGs in jSLE.

### Multiomics analysis of neutrophil metabolome and transcriptome reveals key cellular pathways up- and down-regulated in SLE

We next performed integrated multiomics analysis of neutrophil metabolomics and transcriptomics data in IPA by combining the fold change in gene expression with the fold-change metabolite abundances. The most highly predicted pathways in aSLE vs HA involved a down-regulation of several metabolic processes (including genes and metabolites), including glutamate and glutamine metabolism (adj. *P*-value = 1.26E-12), nucleotide biosynthesis (adj. *P*-value = 1.26E-12), and Warburg effect (adj. *P*-value = 1.26E-08) alongside an up-regulation of immune-related pathways originally identified from transcriptome analysis only (Fig. 8a). On the other hand, both jSLE vs HJ (Fig. 8b) and comparison of aSLE with jSLE (Fig. 8c) revealed key differences primarily involving immune regulatory pathways including IFN-*α/β* (jSLE vs HJ adj. *P*-value = 3.98E-41, aSLE vs jSLE adj. *P*-value = 3.16E-35) and IFN signalling (jSLE vs HJ adj. *P*-value = 1.99E-17, aSLE vs jSLE adj. *P*-value = 5.11E-15), ISGylation signalling pathway (jSLE vs HJ adj. *P*-value = 5.01E-11, aSLE vs jSLE adj. *P*-value = 1.29E-07), along with neutrophil-specific activity pathways, such as neutrophil degranulation (jSLE vs HJ adj. *P*-value = 5.01E-11, aSLE vs jSLE adj. *P*-value = 3.16E-12), NETs (jSLE vs HJ adj. *P*-value = 2.19E-03, aSLE vs jSLE adj. *P*-value = 8.70E-05), and antimicrobial peptides aSLE vs jSLE adj. *P*-value = 3.63E-04. Related to SLE-specific disease context, the SLE in B-cell signalling pathway was also enriched (jSLE vs HJ adj. *P*-value = 1.74E-03, aSLE vs jSLE adj. *P*-value =3.24E-02).

The significant alterations identified in the glutamate and glutamine metabolism pathway in aSLE neutrophils highlights key metabolites and enzymatic steps involved in nitrogen transport, mitochondrial function, and redox balance (Fig. 9a). Central nodes such as L-glutamate (L-Glu; observed) and L-glutamine (L-Gln; predicted) connected multiple reactions involving amino acid transamination likely via GOT2, ammonia handling, and nucleotide cofactor cycling (e.g. NADP⁺/NADPH). Furthermore, the analysis highlighted strong upregulation of type I IFN (IFN-*α/β*) signalling in aSLE neutrophils, evidenced by robust upregulation of several ISGs, such as IFI27, ISG15, RSAD2, and OAS, as well as reduced levels of metabolites ADP and ATP (Fig. 9b). These findings are consistent with a commonly heightened IFN signature observed in both adult and juvenile SLE in this study. An additional downregulated canonical pathway identified in aSLE was the Warburg effect metabolic pathway that illustrates key metabolic reprogramming events, with lower intracellular glucose levels in aSLE leading to lower glycolysis, lactate, NADP, and ATP production. This multiomics approach, incorporating observed changes in gene and metabolite levels, resulted in increased predictive power from the bioinformatics analysis via IPA.

## Discussion

In this study, we have combined metabolomics and transcriptomics techniques to provide the first, multi-omics analysis of neutrophils from adults and children with SLE. Our analyses revealed distinct metabolic and transcriptomic alterations, highlighting unique immune dysregulation in SLE neutrophils compared to healthy individuals, and as a direct comparison between adult and paediatric disease.

Neutrophils primarily generate energy through glycolysis. Under aerobic conditions, neutrophils can utilize oxidative phosphorylation to maximize ATP production, whereas in anaerobic metabolism, pyruvate is reduced into lactate and excreted as a byproduct [33]. This metabolic flexibility becomes particularly crucial under glucose-deprived conditions, allowing neutrophils to sustain their function. In addition to glucose metabolism, short-chain fatty acids, such as acetate, propionate, and butyrate, play key roles in neutrophil activity, supporting processes like migration, chemotaxis, and mitochondrial metabolism [34]. Glutaminolysis can also serve as an alternative energy source when glucose is limited [35]. Notably, increased neutrophil apoptosis has been reported in conditions, such as severe infections and sepsis, where glutamine depletion occurs, a process that can be reversed with glutamine supplementation [36]. Excessive neutrophil apoptosis is a hallmark of SLE [33, 37], and in this study, we observed a metabolic signature with consistently lower levels of glutamate, glutamine, and proline in aSLE and in around 50% of jSLE patients. Intracellular metabolites of glycolysis (glucose, lactate, ATP) were all lower in aSLE compared to HA, with acetate and propionate elevated, suggesting potential metabolic adaptations in response to disease pathology. Other key metabolites, including taurine and NAD were lower in aSLE compared to HA, but higher in jSLE. The stark contrast observed may stem from the significantly higher proportion of participants with active disease (100% in jSLE vs 30% in aSLE) and severe clinical phenotypes among the jSLE cohort and may suggest that an altered metabolic pathway in aSLE neutrophils contributes to their sustained activation, aberrant NETosis, and increased apoptosis.

Prior findings have reported glutathione depletion in circulation in SLE [38] and specifically within T cells [39] attributed to persistent mitochondrial hyperpolarisation. This phenomenon reduces T cell activation, predisposing them to apoptosis and pro-inflammatory necrotic cell death. Mitochondrial hyperpolarisation is closely linked to increased oxidative stress in T cells, evidenced by decreased cysteine levels, suggesting alterations in pathways such as the pentose phosphate pathway (PPP), taurine, thiamine, Gly-Ser-Thr, Cys-Met, and sulphur metabolism [39]. In this study, glutathione depletion was observed exclusively in aSLE neutrophils, alongside cysteine depletion, which may correlate with impaired glutathione metabolism and/or taurine and hypotaurine metabolism. Previous findings have also reported methyl donor depletion, disrupting glutathione regeneration [38]. In line with this, our study observed lower levels of methionine, choline, o-phosphocholine, and phosphocholine, although these did not reach statistical significance in aSLE vs HA. However, we did not observe significant up-regulation of the PPP in our pathway analysis, and in fact the multiomics analysis via IPA predicted the PPP to be down-regulated in aSLE neutrophils based on gene expression (lower TALDO1) and metabolite (ATP, NADP+) abundances. Of the key metabolites NADP+ and NADPH, which are essential for PPP and generation of ROS and NETs in neutrophils [9], only NADP+ showed significant differences in aSLE vs jSLE but not in comparisons with healthy controls. Importantly, we did not collect data on N-acetylcysteine (NAC) treatment among our participants, despite previous studies demonstrating NAC efficacy in SLE patients [39], where glutathione depletion was reversed, leading to improvements in disease activity.

Metabolic pathway analysis revealed similarities between SLE and various diseases in the human blood disease database. In comparison to healthy controls, both aSLE and jSLE shared pathways with schizophrenia, different forms of preeclampsia/eclampsia, malignancies (e.g. colorectal and pancreatic cancer), heart failure, Alzheimer’s disease, metabolic deficiencies (Lipoyltransferase 1, Fumarase, Coenzyme Q10), and infectious states (dengue fever, sepsis). However, no direct SLE pathway was identified, despite overlaps in inflammatory cascades, such as those with early-onset severe pre-eclampsia likely due the presence of anti-phospholipid antibodies or other evident of anti-phospholipid syndrome [40]. The KEGG database lists the SLE pathway (H00080), but it lacks mapped metabolites, instead being dominated by gene lists and thereby not included in the set of pathways available for Metaboanalyst enrichment analysis. This highlights a limitation in current pathway analysis tools, which do not integrate key dysregulated immune-related metabolites and lipoproteins identified in NMR metabolomics studies.

This study also identified underlying transcriptional differences that distinguish jSLE from aSLE. Hierarchical clustering of ISGs and NGPs in isolated neutrophils revealed a comparable upregulation of ISGs across both adult and juvenile SLE groups. This expression pattern aligns with previous literature [2–4], including where a subset of SLE individuals in both age groups exhibited an ‘IFN low’ signature. The results were consistent with hierarchical clustering and the multi-omics canonical pathway comparison between SLE and healthy controls, with IFN-*α/β* and IFN signalling, including IFNγ (only in transcriptomics), emerging as the most significantly activated pathways in SLE, alongside neutrophil degranulation. Interferon-activated SLE neutrophils release pro-inflammatory molecules, including NETs, which contain immunostimulatory DNA that can drive disease progression both intravascularly and through immune complex deposition particularly in LN [1, 4]. The NETting neutrophil is aggravated in a fraction of individuals with SLE with impaired NET degradation and clearance, leading to deposition of circulating NETs, amplifying the interferogenic impact of NETs. Emerging evidence also highlights the role of neutrophils in modulating B cell responses. In humans, activated neutrophils have been shown to express BAFF/BLyS, while murine models demonstrate that neutrophil-derived IFN*α* impairs B cell development and promotes the expression of BAFF and APRIL, key mediators in SLE pathogenesis [41]. These findings align with the prominent enrichment of B cell signalling pathways involving neutrophils, as revealed by the IPA canonical pathway analysis in SLE neutrophils across all ages, consistently in transcriptomics and multi-omics canonical pathways.

While IFN-*α/β* signalling is often dominant in SLE studies, the presence of an IFN*γ* signature in SLE blood samples suggests additional immunological complexity. Notably, several studies have linked the IFN*γ* signature to IFNG expression, specific clinical features, SLE disease activity index (SLEDAI), dsDNA levels, substantial tissue damage particularly in proliferative LN, and inversely correlated with complement (C3 and C4) levels, warranting further investigation into its clinical implications [3, 42]. Individuals with single-nucleotide polymorphisms in IFN*γ* genes are also known to significantly possess susceptibility to SLE [43]. Given its potential role in disease development and progression, the IFN*γ* signature should not be overlooked. A primed neutrophil is also capable of producing ROS under the sole influence of IFN*α* [44] and IFN-α/*β* is involved in NK cell activation [45], whereas IFN*γ* is primarily produced by NK cells and plays a critical role in driving CD4+ T-cell activation, particularly into inflammatory Th17 subsets [46]. Prior *in vitro* studies have demonstrated a delay in neutrophil apoptosis can be induced by IFNα in dose-dependent manner [44] and that both IFNα and IFNγ can induce transcriptional plasticity in healthy neutrophils that direct the immune response beyond simply activation of neutrophil functions [47]. These interactions between type-I and II IFN involving innate and adaptive immune response may contribute to the inflammatory cascade in SLE pathogenesis and warrants further exploration.

A contrasting observation in GOBP analysis provides a fascinating insight into the possibilities of differing pathogenic mechanisms at play in different SLE groups. It is plausible that the cell cycle dysregulation and antiviral/immune response pathways elevated in aSLE compared to organ-system development affected in jSLE were reflected from much more severe clinical presentation in jSLE, where more than 96% of jSLE had biopsy-proven renal involvement, nearly 60% with mucocutaneous manifestations, and 80% presenting with at least one haematological disorder. The prominence of antiviral responses in aSLE raises the question of whether viral infections play a significant role in triggering or exacerbating the antiviral defence in adults compared to children [48]. The identification of cell cycle and chromosome replication pathways in aSLE neutrophils through GOBP analysis is intriguing, considering that neutrophils are terminally differentiated cells that do not undergo cell division in peripheral blood [49, 50]. It has been proposed that ROS-induced NETosis, which requires chromatin dismantling, may be regulated similarly to the initiation of cell division [33, 51]. This process is triggered by the induction of cell cycle regulators, including cyclin-dependent kinases (CDKs), such as CDK1 and CDK2 [51] and also involves CDK4/6 activation, which is known to regulate G1/S phase transition in the cell cycle [50]. NET formation may thus resemble a form of truncated cell cycle re-entry culminating in cytokinesis and chromatin decondensation, mediated by checkpoint kinase 1 under ROS stimulation.

Interestingly, beside the transcriptomic profile in SLE individuals, this study identified an atypical subset of four healthy adults in Thailand displaying an IFN-high signature, which may reflect underlying genetic, environmental, or regional factors influencing baseline IFN activity. People with high IFN levels are at risk of developing autoimmune disease through upregulation of toll-like receptors and the loops of persistent inflammatory cascades [52]. In Thailand, particularly among people in the northeastern provinces and Mekong region, human infection with Asian river fluke *(Opisthorcis viverrini)* is a long-known public health problem [53, 54]. Humans as the host may develop cholangiocarcinoma later in life in those individuals carrying an IFNγ polymorphism [53], with persistent inflammation, host tissue damage, and imbalanced tissue damage–repair mechanism attributed to delayed apoptosis of the parasite, mediated tissue injury (e.g. ROS and protease) by activated neutrophils, and circulating proinflammatory cytokines produced by peripheral blood mononuclear cells (PBMC) contributing to innate immune cells driving systemic inflammation, and neutrophil–*Helicobacter pylori*-mediated liver carcinogenesis [54].

Multiomics analysis identified converging pathological processes underpinning SLE neutrophil immunometabolism dysregulation. Notably, the multi-omics analysis highlighted dysregulation of glutamate and glutamine metabolism, particularly in aSLE. Glutamine is the most abundant non-essential amino acid in the human body (plasma concentration approximately 0.4–0.6 mM), and neutrophils consume more glutamine than other PBMCs, such as monocytes and lymphocytes, particularly under stress [55]. Glutamine uptake supports neutrophil survival by reducing extrinsic apoptosis (via decreased phosphorylation of p38, MAPK, and JNK) and enhancing $O_{2}^{-}$ production, which is critical for NETosis [56]. Additionally, glutamine metabolism sustains mitochondrial function and delays apoptosis through NADPH oxidase activation [35]. Glutamine is converted into glutamate, aspartate, and lactate, fuelling oxidative metabolism through glutaminolysis. Glutamate serves as a key intermediate, generating *α*-ketoglutarate and NADPH to support ROS production during NETosis [33]. Previous *in vitro* metabolomics from our group showed that *in vitro* activated healthy neutrophils contain higher levels of lactate and NADPH, with decreased NAD⁺ and NADP⁺, suggesting a metabolic shift from glycolysis towards the PPP and PRPP pathways to enhance ROS generation [6]. This metabolic shift is echoed in RA neutrophils [9]. However, the results of this study indicate that genes and metabolites of glycolysis, glutamine metabolism, and PPP are lower in aSLE and in a proportion of jSLE neutrophils, distinguishing SLE neutrophil pathology from that seen in RA.

Another key finding from the integrated multiomics pathway enrichment analysis of aSLE was the downregulation of the Warburg effect [57], or aerobic glycolysis. This prediction was driven by lower levels of metabolites in aSLE, including ATP, glucose, lactate, NADH, NAD, and NADP+, rather than changes in expression of glycolysis genes. Contrasting to tumour cells, this study observed aSLE neutrophils downregulating glycolysis under metabolic stress to sustain their function to NETosis and apoptosis. The signature feature of enhanced neutrophil apoptosis in SLE has been consistently reported [33, 37], and our data further support this. Importantly, glycolysis, PPP and glutaminolysis are downregulated during apoptosis but are known to be upregulated during NETosis [36]. Apoptotic priming in neutrophils requires glutamine, glutamate, taurine, and proline depletion, together with glycolytic inhibition, reinforcing the idea that neutrophil fate is tightly coupled to metabolic flux. Notably, other metabolomic studies on apoptotic neutrophils aged 30 h *in vitro* reported reduced levels of taurine, ADP, and ATP, further supporting our findings [58]. Significantly lower glutathione abundance was also observed in the neutrophil metabolome of aSLE patients compared to HA. Notably, reduced levels of methionine, choline, o-phosphocholine, and phosphocholine, as previously reported in SLE neutrophils [38], may contribute to compromised glutathione synthesis. Importantly, glutathione plays a critical role beyond xenobiotic detoxification; it neutralizes hydrogen peroxide (H₂O₂), a potent pro-apoptotic molecule in immune cells. Thus, glutathione depletion in aSLE neutrophils may contribute to the heightened susceptibility to apoptosis via impaired redox homeostasis and excess H₂O₂.

To our knowledge, this is the first study to profile neutrophil metabolism and transcription in both aSLE and jSLE, offering insights into altered neutrophil phenotype in SLE. These findings underscore distinct metabolic and transcriptomics adaptations in neutrophils between aSLE and jSLE, possibly influenced by disease duration, immune system maturity, or differential metabolic demands. A considerable limitation of this study is the participant selection bias, which may have influenced neutrophil isolation and transcriptional profiles. None of the SLE patients were treatment-naïve, and certain medications are known to modulate type I IFN activity and circulating neutrophil levels, potentially affecting the observed gene expression patterns. Future studies incorporating multiple timepoints, e.g. pre-treatment, during active disease, and in remission, would provide a more comprehensive and objective assessment of neutrophil transcriptional dynamics. In addition, future studies could adopt a more stringent inclusion and exclusion criteria such as high disease activity based on SLEDAI-2K and limited to renal involvement or those with high IFN signatures as well as a larger sample size to enhance the power of the omics analysis. A multi-site concerted approach is recommended to compensate for small sample size in this rare but debilitating disease. This is particularly important for juvenile research given the wide variance of symptoms and inherent metabolic variance within children. Validation of the upstream regulator predictions should also be incorporated into future studies, for example, by measuring phosphorylated (active) transcription factor expression by Western blot. We also recognize that this is a multisite study, recruiting participants in the UK and in Thailand. Whilst we did not identify any notable differences in metabolite and gene profiles between South Asian and Caucasian participants, we cannot discount that some of the differences between aSLE (recruited in the UK and Thailand) and jSLE (all recruited in Thailand) could be caused by geographic variations (including diet, environmental exposures such as UV, as well as genetics) rather than differences in adult and paediatric disease. We also acknowledge that whilst our paediatric healthy controls (HJ) were all of Thai ethnicity, our adult healthy controls (HA) were a mix of Caucasian and South Asian ethnicities. Despite these limitations, our findings underscore a robust ISG signature in both aSLE and jSLE, with a more pronounced IFN response in jSLE. Notably, hierarchical clustering revealed a consistent neutrophil gene expression pattern across both SLE subgroups, reinforcing the role of neutrophils in the pathogenesis of SLE. These insights highlight the need for further investigation into the interplay between IFN signalling, neutrophil activation, and disease progression in SLE.

In summary, this study provides the first multiomic analysis of gene expression and metabolism in adult and paediatric SLE, providing a strengthened understanding of the impact of altered neutrophil metabolism and transcription in the pathogenesis of SLE. Understanding these molecular signatures could provide insight into age-related variations in SLE pathophysiology and aid in identifying potential metabolic targets for tailored therapeutic strategies.

## Data availability

All metabolomics data including processing steps, parameters, and metabolite annotations and CRS representative peak selections are deposited in the European Bioinformatics Institute (EBI) open repository for metabolomics (www.ebi.ac.uk/metabolights) with the ID number MTBLS6259 [29]. The raw RNA sequencing data reported in this manuscript have been deposited in the NCBI Gene Expression Omnibus (GEO) [30] and are accessible through GEO Series accession number GSE293224.

## Abbreviations

BH: Benjamini–Hochberg
CDK: cyclin-dependent kinase
CRS: correlation-based scoring
DEG: differentially expressed gene
FC: fold change
FDR: false discovery rate
GOBP: Gene Ontology Biological Process
HA: healthy adult
HJ: healthy juvenile
IC: immune complexes
IFN: interferon
IPA: ingenuity pathway analysis
ISG: interferon-stimulated gene
LN: lupus nephritis
NAC: N-acetylcysteine
NET: neutrophil extracellular trap
NGP: neutrophil granule protein
PCA: principal component analysis
PLS-DA: partial least squares discriminant analysis
RIN: RNA integrity
ROS: reactive oxygen species
SLE: systemic lupus erythematosus
SLEDAI: SLE disease activity index
TMM: trimmed mean of M-values
VIP: Variable importance in the projection
