# Supplementary material for: Multiomics analysis of neutrophils in SLE: insights from adult and paediatric disease
Source: Clin Exp Immunol. 2025 Dec 9;219(1):uxaf077. doi: 10.1093/cei/uxaf077 (PMC12721378; doi:10.1093/cei/uxaf077)
Supplement: uxaf077_Supplementary_Data [file uxaf077_supplementary_data.docx]

**Supplementary Table 1.** Demographic comparisons of clinical characteristics and laboratory parameters between adult (aSLE) and juvenile (jSLE) SLE participants

|  | **Descriptive statistics** | | ***p*-value** |
| --- | --- | --- | --- |
|  | **jSLE (n=28)** | **aSLE (n=13)** |  |
| **Baseline Parameter** | | |  |
| Sex  Female  Male | 22 (78.6)  6 (21.4) | 13 (100)  - | 0.152 |
| Age (year) | 15.5 ± 4 | 41.1 ± 9.6 | - |
| Ethnicity  Asian | 28 (100) | 13 (100) | - |
| Time diagnosed as SLE (years) | 5.1 ± 4.1 | 13 ± 10.2 | <0.001** |
| BMI | 24.7 ± 5.3 | 20.9 ± 2.5 | 0.009* |
| <25 kg/m^2^  25-30 kg/m^2^  >30 kg/m^2^ | 13 (50)  9 (34.6)  4 (15.4) | 13 (100)  -  - | 0.006* |
| **2019 EULAR/ ACR Classification Criteria** | | |  |
| **Clinical criteria met:** | | |  |
| Fever | 21 (75) | 3 (23.1) | 0.003* |
| Mucocutaneous involvement | 16 (57.1) | 3 (23.1) | 0.042* |
| Single type  Mixed type | 6 (37.5)  10 (62.5) | 1 (33.3)  2 (66.7) | 0.891 |
| Joint involvement | 10 (35.7) | 2 (15.4) | 0.228 |
| Serositis | 6 (21.4) | 1 (7.7) | 0.318 |
| Renal disorders  Renal Biopsy  Pure proliferative  Non-proliferative  Mixed proliferative | 27 (96.4)  11 (39.3)  8 (28.6)  3 (10.7) | 11 (91.7)  5 (38.5)  2 (15.4)  3 (23.1) | 0.527  0.456 |
| Neuropsychiatric disorders | 3 (10.7) | - | 0.238 |
| Hematological disorders | 23 (82.1) | 3 (23.1) | 0.001** |
| Leukopenia  Thrombocytopenia  AIHA | 10 (43.5)  6 (26.1)  18 (78.3) | 3 (100)  1 (33.3)  2 (66.6) | 0.589 |
| Single type  Mixed type | 14 (50)  9 (32.1) | 1 (0.8)  2 (15.4) | 0.364 |
| Leukopenia and AIHA  Thrombocytopenia and AIHA  Leukopenia, thrombocytopenia, & AIHA | 4 (44.5)  3 (33.3)  2 (22.2) | 1 (50)  -  1(50) | 1.000 |
| **Autoantibody positivity** | | |  |
| ANA | 28 (100) | 6 (46.2) | <0.001** |
| Homogenous pattern  Fine speckled pattern  Cytoplasmic pattern  Nucleolar pattern | 18 (64.3)  12 (42.9)  2 (0.7)  1 (0.4) | 5 (83.3)  1 (1.7)  2 (3.3)  - | <0.001** |
| Anti-dsDNA | 25 (89.3) | 11 (91.7) | 0.818 |

Value depicted as *mean±SD* otherwise, when necessary, as *median; range*.

Frequency statistic in each parameter also represented in *proportion* in brackets (%).

Significancy level in *p*-value of <0.05(*) and ≤0.001(**)

⤉ eGFR has missing information in jSLE (4) and aSLE (1) population.

∤ To be revised and adjusted with serology status, mucocutaneous involvement and medications history.

**Supplementary Table 1.** **(cont.).**

|  | **Descriptive statistics** | | | | | ***p*-value** | |
| --- | --- | --- | --- | --- | --- | --- | --- |
|  | **jSLE (n=28)** | | **aSLE (n=13)** | | |  | |
| **Autoantibody positivity (cont.)** | | | |  | | | |
| Anti-Sm | 5 (17.9) | 1(7.7) | | | | | 0.261 |
| Anti-phospholipid antibodies |  |  | | | | |  |
| Lupus anticoagulant  Anti-cardiolipin  Anti-β2-GP1 | 15 (53.6)  14 (50)  2 (7.1) | 2(15.4)  -  1(7.7) | | | | | 0.035*  0.018*  0.041* |
| C3  C4 | 28 (100)  20 (71.4) | 7 (53.8)  8 (61.5) | | | | | 0.017*  0.332 |
| Direct Coombs’ | 18 (64.3) | 3 (23.1) | | | | | 1.000 |
| **Laboratory Parameter** | | | | |  | | |
| **Complete Blood Count:** | | | | |  | | |
| Hemoglobin (g/dL) | 9.9 ± 2.7 | | 11.4 ± 3 | | | | 0.704 |
| Hematocrit (cells/mm^3^) | 29.7 ± 7.7 | | 34.5 ± 9.2 | | | | 0.487 |
| White blood cells (cells/mm^3^) | 7,237.1 ± 6,330 | | 5,975.4 ± 2,295.5 | | | | 0.001* |
| Neutrophil (cells/mm^3^) | 3,896.5; 775-27,201 | | 3,030; 1.790-6,190 | | | | 0.417 |
| Lymphocyte (cells/mm^3^) | 1,589.5; 159-2,956 | | 1,470; 450-3,500 | | | | 0.466 |
| Platelet (cells/mm^3^) | 215,178.6 ± 104,844.2 | | 233,692.3 ± 80,045.18 | | | | 0.297 |
| **Urinalysis:** | | | | |  | | |
| Dipstick protein  Negative  1+  2+  3+  4+ | 4 (14.3)  6 (21.4)  5 (17.9)  8 (28.6)  5 (17.9) | | 6 (46.2)  2 (15.4)  3 (23.1)  1 (7.7)  - | | | | 0.098 |
| Creatinine (mg/dL) | 0.6; 0.3-6.4 | | 0.76; 0.3-16 | | | | 0.144 |
| eGFR (ml/min/1.73m^2^ ; Thai CKD-EPI equation)⤉ | 97.3 ± 44.9 | | 81.2 ± 37.6 | | | | 0.508 |
| Microscopic |  | |  | | | |  |
| Pyuria (WBC>5cells/hpf) | 5 (17.9) | | 1 (7.7) | | | | 0.318 |
| Hematuria (RBC>3cells/hpf) | 15 (53.6) | | 2 (15.4) | | | | 0.028* |
| **Disease Activity** | | | | |  | | |
| SLEDAI-2K ∤ | 15.9; 9-30 | | 6; 0-41 | | | <0.001** | |
| Low disease activity (SLEDAI-2K <3) | - | | 5 (38.5) | | | <0.001** | |
| High disease activity (SLEDAI-2K >6) | 28 (100) | | 4 (30.8) | | | <0.001** | |

Value depicted as *mean±SD* otherwise, when necessary, as *median; range*.

Frequency statistic in each parameter also represented in *proportion* in brackets (%).

Significancy level in *p*-value of <0.05(*) and ≤0.001(**)

⤉ eGFR has missing information in jSLE (4) and aSLE (1) population.

∤ To be revised and adjusted with serology status, mucocutaneous involvement and medications history.

**Supplementary Table 2.** Comparative Analysis of SLE Medications in Adult and Juvenile Participants

|  | **Descriptive statistics** | | ***p*-value** | |  |  |
| --- | --- | --- | --- | --- | --- | --- |
|  | **jSLE (n=28)** | **aSLE (n=13)** | |  | | |
| Prednisolone  *Weekly dose (mg)*  *Maximum weekly dose (mg)* | 27 (96.4)  *96; 35-420*  *420; 210-420* | 10 (76.9)  *35; 17.5-210*  *70; 35-560* | | 0.05 | |  |
| Chloroquine | - | 2 (15.4) | | 0.095 | |  |
| Hydroxychloroquine  *Weekly dose (mg)*  *Maximum weekly dose (mg)* | 28 (100)  *1,400; 700-1,400*  *1,400; 700-1,400* | 5 (38.5)  *-; 1,400-2,100*  *1,400; 1,400-2,100* | | <0.001** | |  |
| Cyclophosphamide  *Weekly dose (mg)*  *Maximum weekly dose (mg)* | 12 (42.9)  *500; 162-900*  *201; 125-1,000* | 4 (30.8)  *-*  *-; 800-1,000* | | 0.460 | |  |
| Azathioprine | - | 1 (7.7) | | 0.317 | |  |
| Mycophenolate mofetil  *Weekly dose (mg)*  *Maximum weekly dose (mg)* | 18 (64.3)  *8,750; 1,400-14,000*  *14,000; 7,000-14,000* | 10 (76.9)  *5,250; 3,000-14,000*  *7,000; 3,000-14,000* | | 0.493 | |  |
| Cyclosporine | 1 (3.5) | 1 (7.7) | | 0.569 | |  |
| Methotrexate | 1 (3.5) | 2 (15.4) | | 0.176 | |  |

Weekly treatment dosage (mg) was depicted as *median; range*.

Frequency statistic in each parameter also represented in *proportion* in brackets (%).

Significancy level in *p*-value of <0.05(*) and ≤0.001(**)

NOTE. Due to stricter ethical regulations in the UK, demographic data collection for the UK aSLE cohort (n=5) was more limited than for the Thai participants. However, available data indicate that the UK aSLE group consisted of individuals aged 34-55 years, all females, all Caucasian ethnicities, and 60% had high disease activity (SLEDAI-2K >6). The UK aSLE were prescribed with HCQ (100%), MMF (20%), and prednisolone (20%).

**Supplementary Table 3.** Demographic comparisons of clinical characteristics and laboratory parameters between adult (HA) and juvenile (HJ) healthy control participants

|  | **Descriptive statistics** | | |
| --- | --- | --- | --- |
|  | **HJ (n=18)** | **HA (UK, n=3)** | **HA (Thai, n=13)** |
| **Baseline Parameter** | | |  |
| Sex  Female  Male | 7 (38.9)  11 (61.1) | 2 (66)  1 (34) | 12 (92.3)  1 (7.7) |
| Age (year) | 14 ± 3.6 | 30 ± 5 | 26.6 ± 4.3 |
| Ethnicity  Asian  Caucasian | 18 (100)  - | -  n (100) | n (100) |

| **Upper Bound (ppm)** | **Lower Bound (ppm)** | **Metabolite Bin** | **HMDB Bin** | **CRS Status** |
| --- | --- | --- | --- | --- |
| 0.0869 | 0.0795 | UNKNOWN_1 | _HMDBNULL_1 | N/A |
| 0.1717 | 0.1631 | UNKNOWN_2 | _HMDBNULL_2 | N/A |
| 0.872 | 0.8504 | HYDROXYMETHYLVALERATE_3a | CRS_2-Hydroxy-3-methylpentanoate_HMDB0000317_3a | CRS |
| 0.8962 | 0.872 | UNKNOWN_HYDROXYMETHYLVALERATE_3 | _HMDBNULL_HMDB0000317_3 | N/A |
| 0.9012 | 0.8962 | UNKNOWN_HYDROXYMETHYLVALERATE_HYDROXYBUTYRATE2_4 | CRS_2-Hydroxybutyrate_HMDBNULL_HMDB0000317_HMDB0000008_4 | CRS |
| 0.9058 | 0.9012 | UNKNOWN_5 | _HMDBNULL_5 | N/A |
| 0.9106 | 0.9058 | UNKNOWN_HYDROXYMETHYLVALERATE_6 | _HMDBNULL_HMDB0000317_6 | N/A |
| 0.921 | 0.9106 | UNKNOWN_7 | _HMDBNULL_7 | N/A |
| 0.9368 | 0.921 | ISOLEUCINE_HYDROXYMETHYLVALERATE_8 | _HMDB0000172_HMDB0000317_8 | N/A |
| 0.9466 | 0.9368 | ISOLEUCINE_9 | _HMDB0000172_9 | N/A |
| 0.9613 | 0.9466 | LEUCINE_ISOLEUCINE_10 | _HMDB0000687_HMDB0000172_10 | N/A |
| 0.9704 | 0.9613 | LEUCINE_11a | CRS_Leucine_HMDB0000687_11a | CRS |
| 0.9867 | 0.9704 | LEUCINE_12 | _HMDBNULL_12 | N/A |
| 1.0059 | 0.9867 | VALINE_13 | CRS_Valine_HMDB0000883_13 | CRS |
| 1.0249 | 1.0054 | ISOLEUCINE_14 | CRS_Isoleucine_HMDB0000172_14 | CRS |
| 1.0457 | 1.0368 | VALINE_15 | _HMDB0000883_15 | N/A |
| 1.05 | 1.0457 | PROPIONATE_16 | _HMDB0000237_16 | N/A |
| 1.057 | 1.05 | VALINE_17 | _HMDB0000883_17 | N/A |
| 1.0745 | 1.057 | PROPIONATE_18 | CRS_Propionate_HMDB0000237_18 | CRS |
| 1.1381 | 1.132 | UNKNOWN_20 | _HMDBNULL_20 | N/A |
| 1.1531 | 1.1381 | PROPYLENEGLYCOL_21 | CRS_Propylene glycol_HMDB0001881_21 | CRS |
| 1.159 | 1.1531 | ISOPROPANOL_23a | CRS_Isopropyl alcohol_HMDB0000863_23a | CRS |
| 1.169 | 1.159 | UNKNOWN_ISOPROPANOL_HYDROXYMETHYLVALERATE_23 | _HMDBNULL_HMDB0000863_HMDB0000317_23 | N/A |
| 1.183 | 1.169 | UNKNOWN_ETHANOL_ISOPROPANOL_HYDROXYMETHYLVALERATE_24 | _HMDBNULL_HMDB0000108_HMDB0000863_HMDB0000317_24 | N/A |
| 1.1901 | 1.183 | UNKNOWN_ETHANOL_25a | CRS_Ethanol_HMDBNULL_HMDB0000108_25a | CRS |
| 1.2 | 1.1901 | UNKNOWN_ETHANOL_25 | _HMDBNULL_HMDB0000108_25 | N/A |
| 1.2073 | 1.2 | UNKNOWN_26 | _HMDBNULL_26 | N/A |
| 1.2241 | 1.2073 | UNKNOWN_HYDROXYBUTYRATE3_27 | CRS_3-Hydroxybutyrate_HMDBNULL_HMDB0000011_27 | CRS |
| 1.2407 | 1.2255 | UNKNOWN_28 | _HMDBNULL_28 | N/A |
| 1.2498 | 1.2407 | ISOLEUCINE_29 | _HMDB0000172_29 | N/A |

**Supplementary Table 4.** List of annotated metabolite peaks in 1D ^1^H NMR human neutrophil extracts with the respective chemical shift range, also available via public repository MetaboLights ID: MTBLS 6259

| **Upper Bound (ppm)** | **Lower Bound (ppm)** | **Metabolite Bin** | **HMDB Bin** | **CRS Status** |
| --- | --- | --- | --- | --- |
| 1.2572 | 1.2498 | HYDROXYISOVALERATE2_ISOLEUCINE_30 | CRS_2-Hydroxy-3-methylbutyrate_HMDB0000407_HMDB0000172_30 | CRS |
| 1.2832 | 1.2603 | ISOLEUCINE_31 | _HMDB0000172_31 | N/A |
| 1.3042 | 1.2847 | UNKNOWN_32 | _HMDBNULL_32 | N/A |
| 1.3139 | 1.3046 | ISOLEUCINE_33 | _HMDB0000172_33 | N/A |
| 1.3224 | 1.316 | UNKNOWN_34 | _HMDBNULL_34 | N/A |
| 1.3279 | 1.3224 | LACTATE_35 | CRS_Lactate_HMDB0000190_35 | CRS |
| 1.3301 | 1.3279 | THREONINE_36 | _HMDB0000167_36 | N/A |
| 1.3342 | 1.3301 | UNKNOWN_37 | _HMDBNULL_37 | N/A |
| 1.3376 | 1.3342 | LACTATE_38 | _HMDB0000190_38 | N/A |
| 1.3396 | 1.3376 | THREONINE_39 | _HMDB0000167_39 | N/A |
| 1.356 | 1.3396 | UNKNOWN_HYDROXYMETHYLVALERATE_HYDROXYVALERATE2_40 | _HMDBNULL_HMDB0000317_HMDB0001863_40 | N/A |
| 1.363 | 1.356 | UNKNOWN_41 | _HMDBNULL_41 | N/A |
| 1.38 | 1.363 | UNKNOWN_HYDROXYMETHYLVALERATE_HYDROXYVALERATE2_42 | _HMDBNULL_HMDB0000317_HMDB0001863_42 | N/A |
| 1.437 | 1.38 | HYDROXYVALERATE2_42a | CRS_2-Hydroxyvalerate_HMDB0001863_42a | CRS |
| 1.448 | 1.437 | UNKNOWN_43 | _HMDBNULL_43 | N/A |
| 1.4528 | 1.448 | UNKNOWN_44 | _HMDBNULL_44 | N/A |
| 1.457 | 1.4528 | ISOLEUCINE_LYSINE_SACCHAROPINE_45 | _HMDB0000172_HMDB0000182_HMDB0000279_45 | N/A |
| 1.465 | 1.457 | UNKNOWN_46 | _HMDBNULL_46 | N/A |
| 1.4768 | 1.465 | UNKNOWN_47 | _HMDBNULL_47 | N/A |
| 1.4988 | 1.4768 | ALANINE_ISOLEUCINE_LYSINE_SACCHAROPINE_48 | CRS_Alanine_HMDB0000161_HMDB0000172_HMDB0000182_HMDB0000279_48 | CRS |
| 1.5182 | 1.4988 | UNKNOWN_49 | _HMDBNULL_49 | N/A |
| 1.5401 | 1.5182 | ADIPATE_LYSINE_SACCHAROPINE_50 | _HMDB0040270_HMDB0000182_HMDB0000279_50 | N/A |
| 1.57 | 1.5401 | ADIPATE_SACCHAROPINE_51 | CRS_Diethylhexyl adipate_HMDB0040270_HMDB0000279_51 | CRS |
| 1.6349 | 1.57 | UNKNOWN_52 | _HMDBNULL_52 | N/A |
| 1.7278 | 1.6349 | LEUCINE_ARGININE_53 | _HMDB0000687_HMDB0000517_53 | N/A |
| 1.7374 | 1.7278 | LEUCINE_SACCHAROPINE_ARGININE_LYSINE_54 | _HMDB0000687_HMDB0000279_HMDB0000517_HMDB0000182_54 | N/A |
| 1.767 | 1.7374 | LEUCINE_SACCHAROPINE_LYSINE_55 | CRS_Saccharopine_HMDB0000687_HMDB0000279_HMDB0000182_55 | CRS |
| 1.8095 | 1.7836 | UNKNOWN_56 | _HMDBNULL_56 | N/A |
| 1.8693 | 1.822 | UNKNOWN_57 | _HMDBNULL_57 | N/A |
| 1.9169 | 1.9017 | LYSINE_SACCHAROPINE_ARGININE_58 | _HMDB0000182_HMDB0000279_HMDB0000517_58 | N/A |

| **Upper Bound (ppm)** | **Lower Bound (ppm)** | **Metabolite Bin** | **HMDB Bin** | **CRS Status** |
| --- | --- | --- | --- | --- |
| 1.9225 | 1.9169 | ACETATE_SACCHAROPINE_ARGININE_59 | CRS_Acetate_HMDB0000042_HMDB0000279_HMDB0000517_59 | CRS |
| 1.9414 | 1.9225 | LYSINE_SACCHAROPINE_ARGININE_60 | _HMDB0000182_HMDB0000279_HMDB0000517_60 | N/A |
| 1.982 | 1.972 | UNKNOWN_61 | _HMDBNULL_61 | N/A |
| 2.0094 | 2.0039 | ACETAMIDE_62 | CRS_Acetamide_HMDB0031645_62 | CRS |
| 2.0174 | 2.011 | HOMOSERINE_PYROGLUTAMATE_63 | _HMDB0000719_HMDB0000267_63 | N/A |
| 2.0599 | 2.0174 | GLUTAMATE_HOMOSERINE_PYROGLUTAMATE_64 | _HMDB0000148_HMDB0000719_HMDB0000267_64 | N/A |
| 2.0704 | 2.0599 | GLUTAMATE_HOMOSERINE_PYROGLUTAMATE_65 | _HMDB0000148_HMDB0000719_HMDB0000267_65 | N/A |
| 2.0972 | 2.0704 | UNKNOWN_SACCHAROPINE_66 | _HMDBNULL_HMDB0000279_66 | N/A |
| 2.1195 | 2.0972 | GLUTAMINE_GLUTAMATE_HOMOSERINE_SACCHAROPINE_67 | _HMDB0000641_HMDB0000148_HMDB0000719_HMDB0000279_67 | N/A |
| 2.1379 | 2.1195 | GLUTAMINE_GLUTAMATE_HOMOSERINE_68 | _HMDB0000641_HMDB0000148_HMDB0000719_68 | N/A |
| 2.1421 | 2.1379 | GLUTAMINE_GLUTAMATE_HOMOSERINE_METHIONINE_69 | CRS_ Glutamine _HMDB0000641_HMDB0000148_HMDB0000719_HMDB0000696_69 | CRS |
| 2.1634 | 2.1421 | GLUTAMINE_GLUTAMATE_HOMOSERINE_70 | _HMDB0000641_HMDB0000148_HMDB0000719_70 | N/A |
| 2.1686 | 2.1634 | PROPIONATE_71 | _HMDB0000237_71 | N/A |
| 2.1701 | 2.1686 | GLUTAMINE_PROPIONATE_METHIONINE_72 | _HMDB0000641_HMDB0000237_HMDB0000696_72 | N/A |
| 2.191 | 2.1701 | ADIPATE_PROPIONATE_METHIONINE_73 | _HMDB0040270_HMDB0000237_HMDB0000696_73 | N/A |
| 2.2298 | 2.1967 | UNKNOWN_METHIONINE_74 | _HMDBNULL_HMDB0000696_74 | N/A |
| 2.2385 | 2.2298 | ACETONE_75 | CRS_ Acetone _HMDB0001659_75 | CRS |
| 2.247 | 2.241 | UNKNOWN_76 | _HMDBNULL_76 | N/A |
| 2.255 | 2.247 | UNKNOWN_77 | _HMDBNULL_77 | N/A |
| 2.2618 | 2.255 | UNKNOWN_78 | _HMDBNULL_78 | N/A |
| 2.2674 | 2.2618 | ACETOACETATE_VALINE_79 | CRS_Acetoacetate_HMDB0000060_HMDB0000883_79 | CRS |
| 2.3053 | 2.2706 | VALINE_80 | _HMDB0000883_80 | N/A |
| 2.3117 | 2.306 | UNKNOWN_81 | _HMDBNULL_81 | N/A |
| 2.3642 | 2.3117 | GLUTAMATE_82 | _HMDB0000148_82 | N/A |
| 2.3716 | 2.367 | GLUTAMATE_HYDROXYISOVALERATE2_SACCHAROPINE_83 | CRS_Glutamate_HMDB0000148_HMDB0000407_HMDB0000279_83 | CRS |
| 2.3836 | 2.3747 | GLUTAMATE_PYROGLUTAMATE_SACCHAROPINE_84 | _HMDB0000148_HMDB0000267_HMDB0000279_84 | N/A |
| 2.421 | 2.3917 | PYROGLUTAMATE_SACCHAROPINE_85 | _HMDB0000267_HMDB0000279_85 | N/A |
| 2.5003 | 2.4262 | PYROGLUTAMATE_GLUTAMINE_86 | _HMDB0000267_HMDB0000641_86 | CRS |
| 2.5303 | 2.5017 | PYROGLUTAMATE_87 | CRS_ Pyroglutamate _HMDB0000267_87 | CRS |
| 2.5508 | 2.5303 | GLUTATHIONE_UNKNOWN_88 | _HMDB0000125_HMDBNULL_88 | N/A |
| 2.5938 | 2.5517 | GLUTATHIONE_89 | CRS_Glutathione_HMDB0000125_89 | CRS |

| **Upper Bound (ppm)** | **Lower Bound (ppm)** | **Metabolite Bin** | **HMDB Bin** | **CRS Status** | |
| --- | --- | --- | --- | --- | --- |
| 2.5993 | 2.595 | UNKNOWN_90 | _HMDBNULL_90 | | N/A |
| 2.6541 | 2.5993 | UNKNOWN_METHIONINE_91 | _HMDBNULL_HMDB0000696_91 | | N/A |
| 2.6676 | 2.6568 | METHIONINE_ASPARTATE_UNKNOWN_92 | _HMDB0000696_HMDB0000191_HMDBNULL_92 | | N/A |
| 2.699 | 2.6676 | ASPARTATE_93 | CRS_Aspartate_HMDB0000191_93 | | CRS |
| 2.715 | 2.699 | UNKNOWN_94 | _HMDBNULL_94 | | N/A |
| 2.73 | 2.715 | UNKNOWN_95 | _HMDBNULL_95 | | N/A |
| 2.735 | 2.7313 | DIMETHYLAMINE_96 | CRS_Dimethylamine_HMDB0000087_96 | | CRS |
| 2.7544 | 2.738 | UNKNOWN_97 | _HMDBNULL_97 | | N/A |
| 2.767 | 2.7544 | UNKNOWN_SARCOSINE_98 | CRS_Sarcosine_HMDBNULL_HMDB0000271_98 | | CRS |
| 2.779 | 2.767 | UNKNOWN_99 | _HMDBNULL_99 | | N/A |
| 2.797 | 2.779 | UNKNOWN_100 | _HMDBNULL_100 | | N/A |
| 2.8097 | 2.7997 | ASPARTATE_101 | _HMDB0000191_101 | | N/A |
| 2.8251 | 2.8174 | UNKNOWN_102 | _HMDBNULL_102 | | N/A |
| 2.835 | 2.8251 | ASPARTATE_103 | _HMDB0000191_103 | | N/A |
| 2.884 | 2.8424 | ASPARAGINE_104 | CRS_Asparagine_HMDB0000168_104 | | CRS |
| 2.9707 | 2.913 | ASPARAGINE_GLUTATHIONE_105 | _HMDB0000168_HMDB0000125_105 | | N/A |
| 3.0268 | 2.9707 | UNKNOWN_106 | _HMDBNULL_106 | | N/A |
| 3.0432 | 3.0268 | LYSINE_107 | CRS_Lysine_HMDB0000182_107 | | CRS |
| 3.0819 | 3.044 | SACCHAROPINE_108 | _HMDB0000279_108 | | N/A |
| 3.1756 | 3.1144 | PHENYLALANINE_109 | _HMDB0000159_109 | | N/A |
| 3.185 | 3.178 | UNKNOWN_110 | _HMDBNULL_110 | | N/A |
| 3.2025 | 3.1875 | UNKNOWN_111 | _HMDBNULL_111 | | N/A |
| 3.2129 | 3.2046 | CHOLINE_112 | CRS_Choline_HMDB0000097_112 | | CRS |
| 3.2211 | 3.2129 | UNKNOWN_113 | _HMDBNULL_113 | | N/A |
| 3.2284 | 3.2211 | OPHOSPHOCHOLINE_114 | CRS_Phosphorylcholine_HMDB0001565_114 | | CRS |
| 3.2354 | 3.2284 | UNKNOWN_115 | _HMDBNULL_115 | | N/A |
| 3.2541 | 3.2354 | GLUCOSE_116 | _HMDB0000122_116 | | N/A |
| 3.2608 | 3.2541 | ARGININE_TAURINE_117 | CRS_Taurine_HMDB0000517_HMDB0000251_117 | | CRS |
| 3.2653 | 3.2608 | GLUCOSE_118 | _HMDB0000122_118 | | N/A |
| 3.2797 | 3.2653 | ARGININE_TAURINE_119 | _HMDB0000517_HMDB0000251_119 | | N/A |
| 3.2993 | 3.2821 | MYOINOSITOL_120 | _HMDB0000211_120 | | N/A |
| 3.3478 | 3.3001 | UNKNOWN_PROLINE_121 | CRS_Proline_HMDBNULL_HMDB0000162_121 | | CRS |

| **Upper Bound (ppm)** | **Lower Bound (ppm)** | **Metabolite Bin** | **HMDB Bin** | **CRS Status** |
| --- | --- | --- | --- | --- |
| 3.365 | 3.349 | UNKNOWN_PROLINE_MANNOSE_122 | CRS_Mannose_HMDBNULL_HMDB0000162_HMDB0000169_122 | CRS |
| 3.3914 | 3.365 | UNKNOWN_MANNOSE_123 | _HMDBNULL_HMDB0000169_123 | N/A |
| 3.4107 | 3.3914 | GLUCOSE_124 | _HMDB0000122_124 | N/A |
| 3.4358 | 3.4107 | TAURINE_GLUCOSE_125 | _HMDB0000251_HMDB0000122_125 | N/A |
| 3.453 | 3.443 | UNKNOWN_126 | _HMDBNULL_126 | N/A |
| 3.512 | 3.4576 | GLUCOSE_127 | _HMDB0000122_127 | N/A |
| 3.52 | 3.512 | UNKNOWN_128 | _HMDBNULL_128 | N/A |
| 3.53928 | 3.52 | MYOINOSITOL_GLUCOSE_129 | _HMDB0000211_HMDB0000122_129 | N/A |
| 3.558 | 3.53928 | GLUCOSE_GLYCEROL_130 | _HMDB0000122_HMDB0000131_130 | N/A |
| 3.564 | 3.558 | UNKNOWN_131 | _HMDBNULL_131 | N/A |
| 3.5783 | 3.5644 | GLYCEROL_132 | _HMDB0000131_132 | N/A |
| 3.6002 | 3.579 | THREONINE_133 | CRS_Threonine_HMDB0000167_133 | CRS |
| 3.6057 | 3.6002 | UNKNOWN_134 | _HMDBNULL_134 | N/A |
| 3.62 | 3.6097 | VALINE_MYOINOSITOL_SACCHAROPINE_135 | _HMDB0000883_HMDB0000211_HMDB0000279_135 | N/A |
| 3.6294 | 3.6251 | MYOINOSITOL_SACCHAROPINE_136 | CRS_ Myoinositol _HMDB0000211_HMDB0000279_136 | CRS |
| 3.6424 | 3.6392 | MYOINOSITOL_137 | _HMDB0000211_137 | N/A |
| 3.6507 | 3.6424 | GLYCEROL_138 | CRS_ Glycerol _HMDB0000131_138 | CRS |
| 3.6585 | 3.6507 | UNKNOWN_139 | _HMDBNULL_139 | N/A |
| 3.6674 | 3.6585 | GLYCEROL_UNKNOWN_140 | _HMDB0000131_HMDBNULL_140 | N/A |
| 3.6803 | 3.6704 | ISOLEUCINE_141 | _HMDB0000172_141 | N/A |
| 3.7255 | 3.7025 | GLUCOSE_142 | _HMDB0000122_142 | N/A |
| 3.751 | 3.7255 | GLUCOSE_UNKNOWN_LEUCINE_143 | _HMDB0000122_HMDBNULL_HMDB0000687_143 | N/A |
| 3.7538 | 3.751 | LYSINE_GLUCOSE_LEUCINE_144 | _HMDB0000182_HMDB0000122_HMDB0000687_144 | N/A |
| 3.7578 | 3.7538 | GLUCOSE_HOMOSERINE_LYSINE_GLUTAMATE_145 | _HMDB0000122_HMDB0000719_HMDB0000182_HMDB0000148_145 | N/A |
| 3.7718 | 3.7578 | GLUCOSE_HOMOSERINE_GLUTAMATE_146 | _HMDB0000122_HMDB0000719_HMDB0000148_146 | N/A |
| 3.7782 | 3.7718 | ARGININE_GLUCOSE_GLUTAMATE_147 | _HMDB0000517_HMDB0000122_HMDB0000148_147 | N/A |
| 3.7834 | 3.7782 | ARGININE_148 | CRS_ Arginine _HMDB0000517_148 | CRS |
| 3.7872 | 3.7834 | ARGININE_GLUTATHIONE_HOMOSERINE_149 | _HMDB0000517_HMDB0000125_HMDB0000719_149 | N/A |
| 3.8188 | 3.7872 | ALANINE_HOMOSERINE_150 | CRS_Homoserine _HMDB0000161_HMDB0000719_150 | CRS |
| 3.8309 | 3.823 | GLUCOSE_151 | CRS_Glucose_HMDB0000122_151 | CRS |
| 3.8649 | 3.8309 | GLUCOSE_HOMOSERINE_SERINE_152 | _HMDB0000122_HMDB0000719_HMDB0000187_152 | N/A |
| 3.891 | 3.878 | GLUCOSE_153 | _HMDB0000122_153 | N/A |

| **Upper Bound (ppm)** | **Lower Bound (ppm)** | **Metabolite Bin** | **HMDB Bin** | **CRS Status** |
| --- | --- | --- | --- | --- |
| 3.8994 | 3.891 | ASPARTATE_GLUCOSE_154 | _HMDB0000191_HMDB0000122_154 | N/A |
| 3.9126 | 3.9061 | GLUCOSE_ASPARTATE_155 | _HMDB0000122_HMDB0000191_155 | N/A |
| 3.9473 | 3.931 | SERINE_UNKNOWN_156 | _HMDB0000187_HMDBNULL_156 | N/A |
| 3.9641 | 3.9528 | SERINE_157 | CRS_Serine _HMDB0000187_157 | CRS |
| 3.9902 | 3.9641 | UNKNOWN_157a | _HMDBNULL_157a | N/A |
| 4.0213 | 3.9904 | ASPARAGINE_SERINE_PHENILALANINE_158 | _HMDB0000168_HMDB0000187_HMDB0000159_158 | N/A |
| 4.0585 | 4.0213 | CYSTEINE_159 | CRS_Cysteine _HMDB0000574_159 | CRS |
| 4.0736 | 4.0611 | MYOINOSITOL_160 | _HMDB0000211_160 | N/A |
| 4.0967 | 4.0736 | PROLINE_161 | _HMDB0000162_161 | N/A |
| 4.1306 | 4.0972 | LACTATE_162 | _HMDB0000190_162 | N/A |
| 4.164 | 4.147 | UNKNOWN_163 | _HMDBNULL_163 | N/A |
| 4.208 | 4.173 | PYROGLUTAMATE_164 | _HMDB0000267_164 | N/A |
| 5.2443 | 5.2325 | GLUCOSE_165 | _HMDB0000122_165 | N/A |
| 5.9546 | 5.9382 | GTP_166 | _HMDB0001273_166 | N/A |
| 6.0041 | 5.9679 | UNKNOWN_167 | _HMDBNULL_167 | N/A |
| 6.1462 | 6.1402 | ATP_ADP_168 | _HMDB0000538_HMDB0001341_168 | N/A |
| 6.1548 | 6.1462 | ATP_ADP_AMP_IMP_169 | _HMDB0000538_HMDB0001341_HMDB0000045_HMDB0002271_169 | N/A |
| 6.1629 | 6.1548 | IMP_AMP_ATP_170 | _HMDB0002271_HMDB0000045_HMDB0000538_170 | N/A |
| 6.8069 | 6.7997 | UNKNOWN_171 | _HMDBNULL_171 | N/A |
| 6.872 | 6.812 | UNKNOWN_172 | _HMDBNULL_172 | N/A |
| 6.9189 | 6.8946 | ACETAMINOPHEN_173 | _HMDB0001859_173 | N/A |
| 7.0535 | 6.9145 | UNKNOWN_174 | _HMDBNULL_174 | N/A |
| 7.0845 | 7.0758 | HISTIDINE_175 | _HMDB0000177_175 | N/A |
| 7.1739 | 7.0845 | UNKNOWN_176 | _HMDBNULL_176 | N/A |
| 7.1988 | 7.187 | ACETAMINOPHEN_177 | CRS_ Acetaminophen _HMDB0001859_177 | CRS |
| 7.2087 | 7.1988 | UNKNOWN_178 | _HMDBNULL_178 | N/A |
| 7.259 | 7.2185 | PHENYLALANINE_UNKNOWN_179 | _HMDB0000159_HMDBNULL_179 | N/A |
| 7.273 | 7.259 | UNKNOWN_180 | _HMDBNULL_180 | N/A |
| 7.286 | 7.273 | UNKNOWN_181 | _HMDBNULL_181 | N/A |
| 7.3786 | 7.286 | PHENYLALANINE_UNKNOWN_182 | CRS_ Phenylalanine _HMDB0000159_HMDBNULL_182 | CRS |
| 7.3953 | 7.3786 | PHENYLALANINE_183 | _HMDB0000159_183 | N/A |

| **Upper Bound (ppm)** | **Lower Bound (ppm)** | **Metabolite Bin** | **HMDB Bin** | **CRS Status** |
| --- | --- | --- | --- | --- |
| 7.415 | 7.3953 | UNKNOWN_184 | _HMDBNULL_184 | N/A |
| 7.434 | 7.415 | PHENYLALANINE_185 | _HMDB0000159_185 | N/A |
| 7.478 | 7.458 | UNKNOWN_186 | _HMDBNULL_186 | N/A |
| 7.5078 | 7.4824 | BENZOATE_INDOLELACTATE_187 | CRS_Benzoate_HMDB0001870_HMDB0000671_187 | CRS |
| 7.5302 | 7.5078 | BENZOATE_188 | _HMDB0001870_188 | N/A |
| 7.5583 | 7.5382 | BENZOATE_189 | _HMDB0001870_189 | N/A |
| 7.62 | 7.605 | UNKNOWN_190 | _HMDBNULL_190 | N/A |
| 7.635 | 7.62 | UNKNOWN_191 | _HMDBNULL_191 | N/A |
| 7.689 | 7.667 | UNKNOWN_192 | _HMDBNULL_192 | N/A |
| 7.7515 | 7.716 | INDOLELACTATE_193 | _HMDB0000671_193 | N/A |
| 7.8268 | 7.7791 | UNKNOWN_194 | _HMDBNULL_194 | N/A |
| 7.8365 | 7.8268 | HISTIDINE_195 | CRS_ Histidine _HMDB0000177_195 | CRS |
| 7.8494 | 7.8365 | UNKNOWN_196 | _HMDBNULL_196 | N/A |
| 7.8599 | 7.8494 | BENZOATE_UNKNOWN_197 | _HMDB0001870_HMDBNULL_197 | N/A |
| 7.8875 | 7.8599 | BENZOATE_198 | CRS_ Indolelactate _HMDB0001870_198 | CRS |
| 7.9714 | 7.95 | UNKNOWN_199 | _HMDBNULL_199 | N/A |
| 8.052 | 8.027 | UNKNOWN_200 | _HMDBNULL_200 | N/A |
| 8.1223 | 8.119 | UNKNOWN_201 | _HMDBNULL_201 | N/A |
| 8.1475 | 8.1429 | GTP_202 | CRS_Guanosine triphosphate_HMDB0001273_202 | CRS |
| 8.1517 | 8.1488 | NADP_203 | _HMDB0000217_203 | N/A |
| 8.1567 | 8.153 | UNKNOWN_204 | _HMDBNULL_204 | N/A |
| 8.1817 | 8.1781 | NAD_205 | CRS_NAD_HMDB0000902_205 | CRS |
| 8.1921 | 8.184 | NADP_206 | CRS_NADP_HMDB0000217_206 | CRS |
| 8.2006 | 8.1945 | NAD_NADP_207 | _HMDB0000902_HMDB0000217_207 | N/A |
| 8.2099 | 8.2052 | NAD_NADP_208 | _HMDB0000902_HMDB0000217_208 | N/A |
| 8.2187 | 8.2139 | IMP_NAD_209 | CRS_Imidazolepropionate_HMDB0002271_HMDB0000902_209 | CRS |
| 8.2397 | 8.2352 | UNKNOWN_NADH_210 | CRS_NADH_HMDBNULL_HMDB0001487_210 | CRS |
| 8.2742 | 8.2719 | ATP_211 | _HMDB0000538_211 | N/A |
| 8.2777 | 8.2742 | ADP_AMP_ATP_212 | CRS_ADP_HMDB0001341_HMDB0000045_HMDB0000538_212 | CRS |
| 8.323 | 8.313 | UNKNOWN_213 | _HMDBNULL_213 | N/A |
| 8.4345 | 8.43 | NAD_214 | _HMDB0000902_214 | N/A |

| **Upper Bound (ppm)** | **Lower Bound (ppm)** | **Metabolite Bin** | **HMDB Bin** | **CRS Status** |
| --- | --- | --- | --- | --- |
| 8.4626 | 8.4562 | FORMATE_215 | CRS_Formate_HMDB0000142_215 | CRS |
| 8.5543 | 8.5407 | ATP_ADP_216 | CRS_Adenosine triphosphate_HMDB0000538_HMDB0001341_216 | CRS |
| 8.5902 | 8.5858 | UNKNOWN_217 | _HMDBNULL_217 | N/A |
| 8.601 | 8.594 | UNKNOWN_218 | _HMDBNULL_218 | N/A |
| 8.6185 | 8.6135 | UNKNOWN_AMP_ATP_219 | CRS_Adenosine monophosphate_HMDBNULL_HMDB0000045_HMDB0000538_219 | CRS |
| 8.71 | 8.696 | UNKNOWN_220 | _HMDBNULL_220 | N/A |
| 8.8479 | 8.8265 | NAD_221 | _HMDB0000902_221 | N/A |

**Supplementary Table 5 – Significant metabolites identified by univariate analysis of aSLE vs HA neutrophils.**

| **Metabolite (Bin)** | **BH *p*-value** |
| --- | --- |
| Acetaminophen | 0.034 |
| Acetate | 0.035 |
| Alanine | 0.027 |
| AMP | 0.027 |
| Arginine | 0.027 |
| ATP/ ADP | 0.034 |
| Glutamate | 0.034 |
| Glutathione | 0.031 |
| GTP | 0.027 |
| Homoserine | 0.045 |
| Lactate | 0.034 |
| Methionine | 0.034 |
| Myoinositol | 0.034 |
| NADH | 0.027 |
| Phenylalanine | 0.027 |
| Propionate | 0.027 |
| Taurine | 0.027 |
| Threonine | 0.027 |


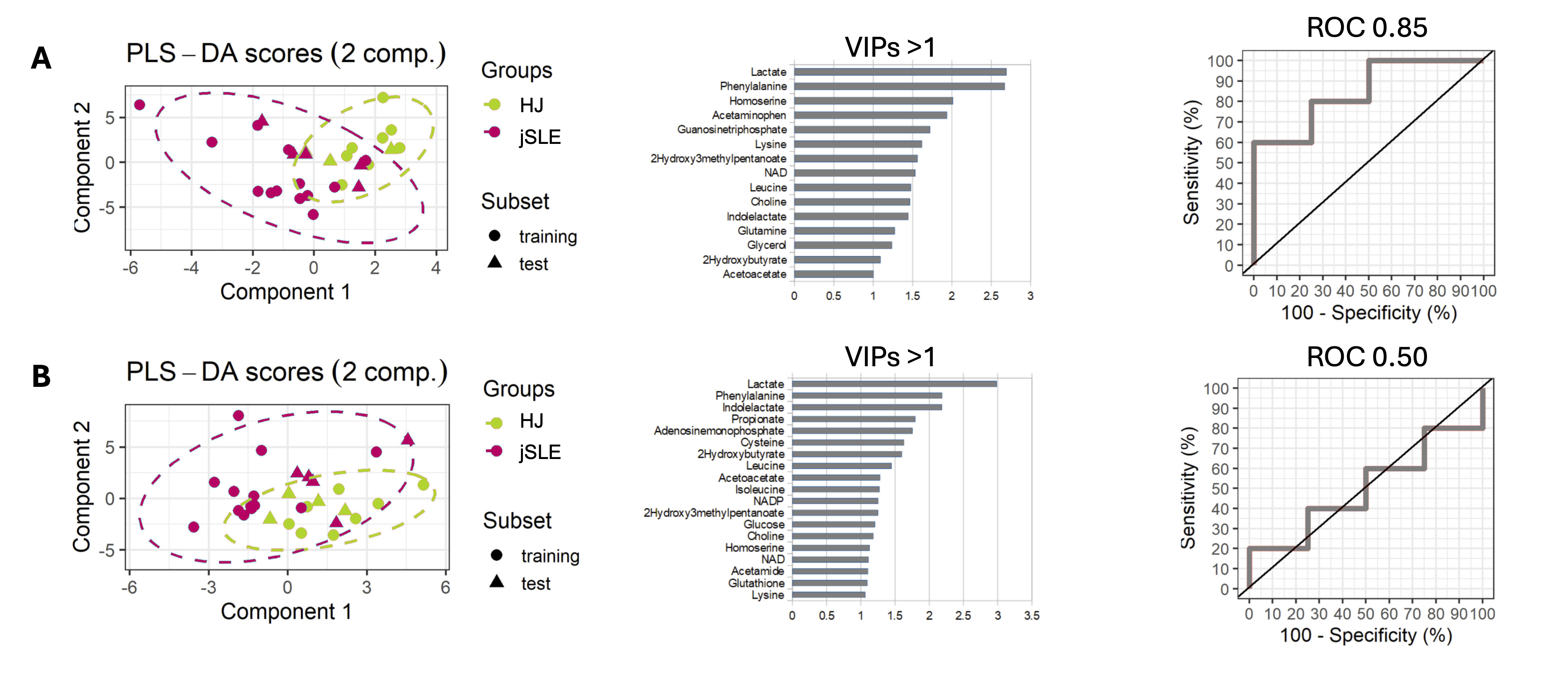


**Supplementary Figure 1. PLS-DA models for jSLE vs HJ neutrophil metabolites.** PLS-DA models were extremely varied in classification metrics, with ROCs between (A) 0.85 and (B) 0.50.


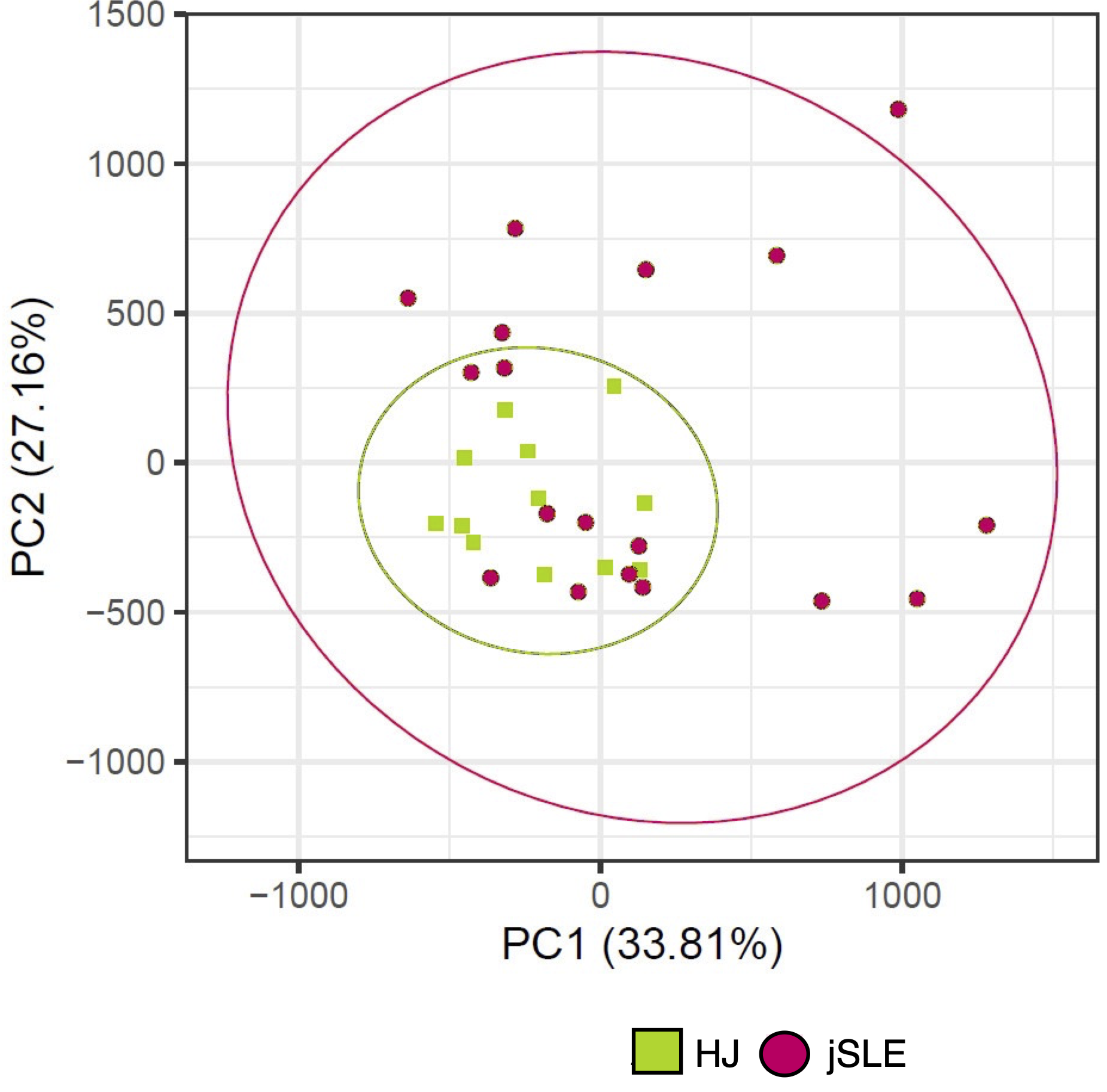


**Supplementary Figure 2. Non-supervised PCA plot built using only VIPs>1 from the PLS-DA model with ROC 0.85.**


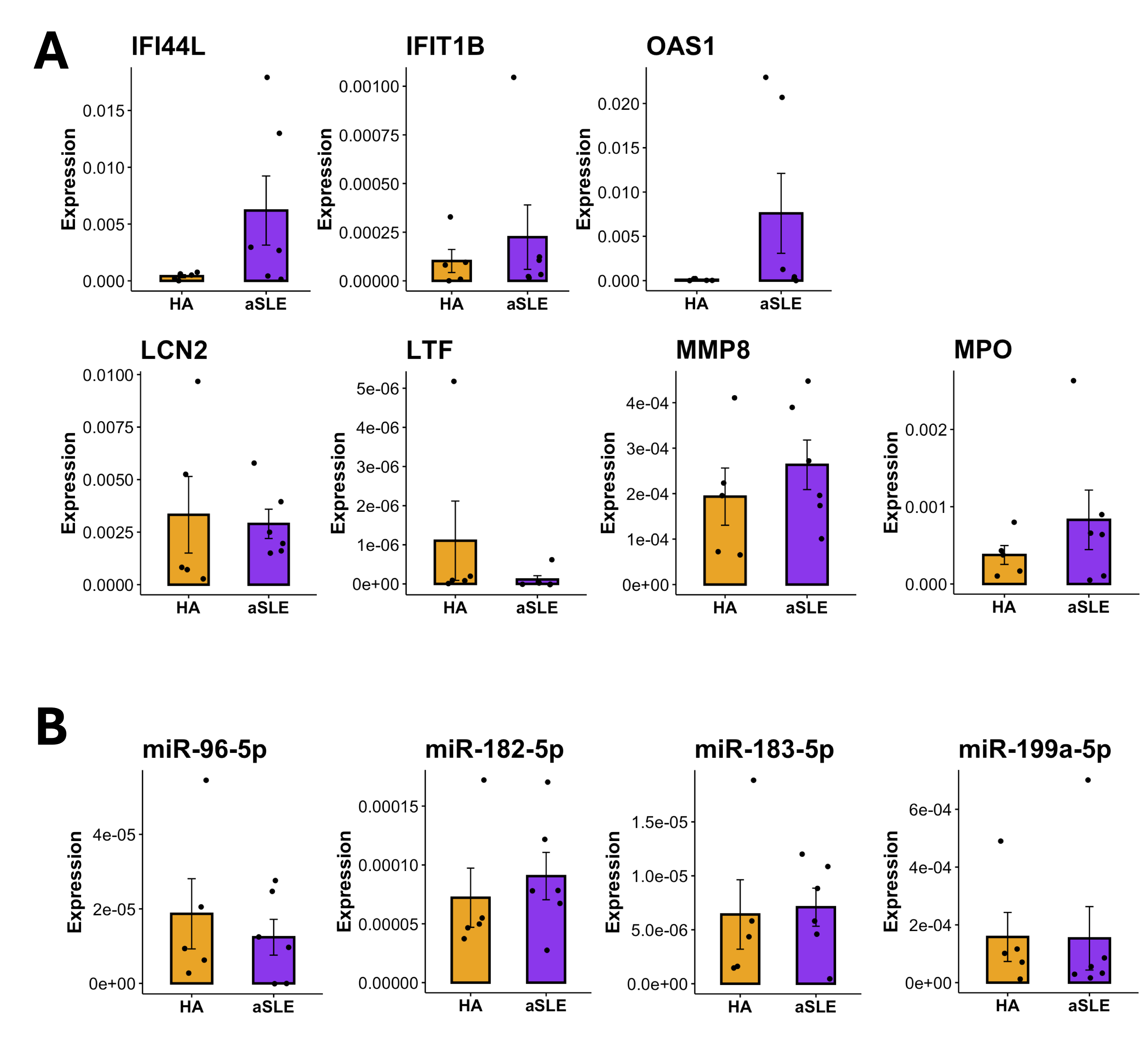


**Supplementary Figure 3. Measurement of neutrophil gene expression by qPCR.** (A) Measurement of ISGs (IFI44L, IFIT1B, OAS1) and neutrophil marker genes (LCN2, LTF, MMP8, MPO) in aSLE and HA neutrophils. (B) Measurement of microRNAs (miR-96-5p, miR-182-5p, miR-183-5p, miR-199-5p) in aSLE and HA neutrophils.


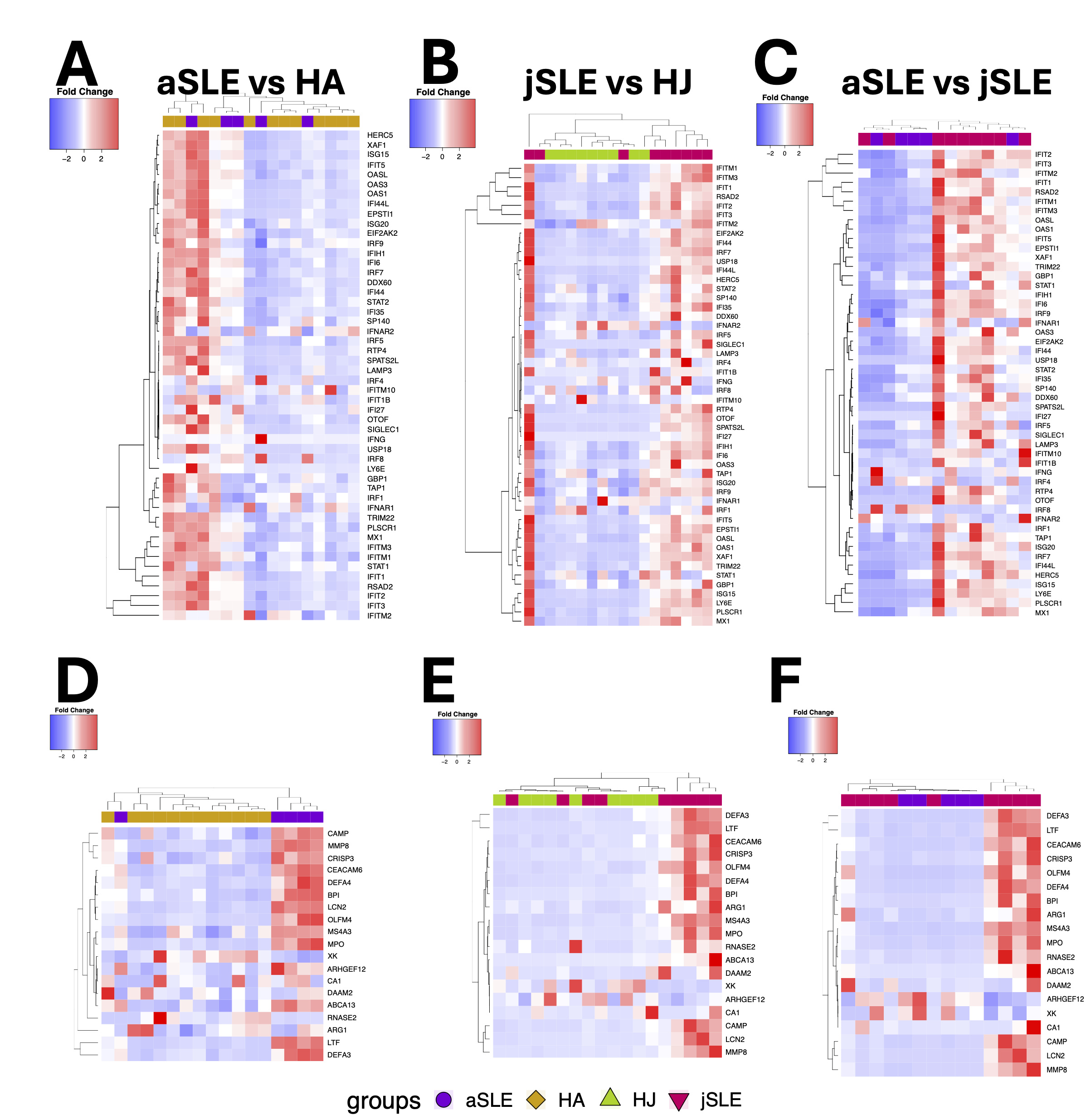


**Supplementary Figure 4. Heatmap analysis of neutrophil gene expression.** ISGs (A-C) and neutrophil marker genes (D-F) expressed in aSLE vs HA, jSLE vs HJ and aSLE vs jSLE neutrophils.
